# Culture Optimization of the IPEC–J2 Piglet Jejunum Cell Line for Applications in Infant Nutrition Research

**DOI:** 10.1002/mnfr.70540

**Published:** 2026-06-25

**Authors:** Francesca Bietto, Myriam M.‐L. Grundy, Elena Arranz, Sandra Wiart‐Letort, Eva Rath, Alice J. Lucey, Linda Giblin

**Affiliations:** ^1^ Teagasc Food Research Centre Fermoy Ireland; ^2^ School of Food and Nutritional Sciences University College Cork Cork Ireland; ^3^ INRAE, French National Research Institute for Agriculture, Food and Environment UMR PEGASE, Institut Agro Saint Gilles France; ^4^ Sección Departamental de Ciencias de la Alimentación Universidad Autónoma de Madrid (UAM) Madrid Spain; ^5^ Institute of Food Science Research CIAL (CSIC‐UAM) Madrid Spain; ^6^ Chair of Nutrition and Immunology, School of Life Sciences Technische Universität München Freising Germany

**Keywords:** amino acid transport, cell differentiation, in vitro digestion, infant milk formula, INFOGEST, IPEC–J2, nutrient absorption, tight junctions, transepithelial electrical resistance (TEER)

## Abstract

The intestinal porcine epithelial cell line (IPEC–J2) is a nontransformed jejunal model derived from unsuckled piglets. It is increasingly used in gut physiology research, yet its application to nutrient absorption remains underexplored. Four media types were assessed for their ability to support IPEC–J2 barrier maturation and permeability: Dulbecco's modified Eagle medium/Ham's F‐12 with 10% fetal bovine serum (FBS10), 10% porcine serum (PS10), 5% porcine serum (PS5) supplemented with Wnt3a‐R‐spondin‐Noggin conditioned medium (PS5+L‐WRN), and PS5 with epidermal growth factor and insulin‐transferrin‐selenium (PS5+EGF+ITS). Culture medium influenced epithelial phenotype, with more than a 30‐fold range in transepithelial electrical resistance (TEER), which plateaued by day 12. PS10 supported a balanced profile, characterized by intermediate TEER (2395.00 ± 485.51 Ω* × *cm^2^), lowest lactulose and mannitol permeability (*p* < 0.05), high lactate dehydrogenase activity (*p* < 0.05 vs. PS5+L‐WRN and FBS10), enhanced membrane localization of tight junction proteins (*p* < 0.05 vs. PS5+EGF+ITS), and reduced fibroblast marker S100A4 (*p* < 0.05 vs. PS5+EGF+ITS). Treatment with digested infant milk formula did not compromise barrier integrity in PS10‐cultured IPEC‐J2 monolayers and enabled transport of free amino acids to the basolateral compartment. These findings support IPEC‐J2 as an alternative to Caco‐2 cells for applications in infant nutrition research in vitro.

## Graphical abstract

IPEC–J2 cell line is derived from piglet jejunal cells. Four different culture media were assessed for their ability to support IPEC‐J2 differentiation into an in vitro gut barrier. PS10 media proved most successful. Once differentiated with PS10, the IPEC‐J2 barrier was sufficiently robust for treatment with digested infant food. This demonstrates the use of this cell line for food absorption studies in Infant Nutrition Research.

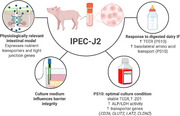

## 1 Introduction

The intestinal epithelium plays a central role in nutrient absorption, barrier integrity, and immune signaling within the gastrointestinal tract. It acts as a selective interface between the external environment and the systemic circulation, facilitating the uptake of nutrients while restricting the translocation of antigens, toxins, and pathogens [1]. This barrier is maintained by a highly organized layer of polarized epithelial cells connected by tight junctions, including zonula occludens‐1 (ZO1), occludin (OCLN), and claudins (CLDN‐1/2/4) [2]. The apical surface is coated with mucins, such as MUC1 (transmembrane) and MUC2 (secreted), which contribute to the protective mucus layer [3]. Embedded within the epithelial membrane are nutrient transporters that mediate the selective uptake of carbohydrates, peptides, amino acids (AA), and lipids [4].

Due to the complexity, cost, and ethical constraints of in vivo studies, in vitro models of intestinal epithelial cells are often the first choice for investigating nutrient absorption, gut function, and responses to dietary or microbial stimuli [5]. Among these, the human‐derived Caco‐2 cell line is the most commonly used model. However, Caco‐2 cells are tumorigenic, of adult origin, and have a low permeability similar to colon [6]. Therefore, their phenotype does not accurately recapitulate the morphology, permeability, or immune responsiveness of the small intestinal epithelium [7]. Transepithelial electrical resistance (TEER), a widely used measure of barrier integrity [8], is typically much higher in Caco‐2 monolayers than in vivo values, highlighting their limited physiological relevance ( > 1000 Ω* × *cm^2^ [9, 10] vs. ∼50–100 Ω* × *cm^2^ [8]). These limitations are particularly important in the context of infant nutrition, where the intestinal barrier is inherently more permeable [11, 12, 13].

Pigs are recognized as a valuable translational model for human gastrointestinal physiology due to similarities in intestinal morphology, nutrient transport, and developmental processes. Neonatal piglets, in particular, closely resemble human infants in terms of gut structure and function [14, 15], supporting their use in pediatric nutrition research [16]. In particular, the gastrointestinal tract of a 3–4 week old piglet closely mimics that of the 3‐month‐old human infant [17]

In this context, the intestinal porcine enterocyte cell line IPEC–J2, isolated from the jejunum of an unsuckled neonatal piglet [18], offers several advantages. It is non‐transformed and is morphologically and functionally similar to primary small intestinal epithelial cells. If grown on a permeable support for 14 days, IPEC–J2 cells grow into tightly adherent, columnar epithelial monolayers that exhibit clear apical‐basal polarity, forming dense brush borders composed of microvilli [19, 20, 21, 22]. The differentiated nature of this epithelial barrier is further supported by the expression of tight junction proteins [23], mucins [7, 24], and nutrient transporters [25, 26, 27, 28]. This broad expression of nutrient transporters makes IPEC–J2 a valuable model for investigating intestinal nutrient absorption and the bioavailability of dietary components. Furthermore, IPEC–J2 cells function as an immunocompetent epithelial model, expressing cytokines, chemokines, toll‐like receptors, and β‐defensins [23, 29]. This makes them a useful system for studying immune‐epithelial interactions and the impact of diet on intestinal immunity.

In terms of metabolism, IPEC–J2 cells rely on both oxidative phosphorylation and glycolysis [20], but will favor glycolysis upon reaching confluence [30]. The capacity to utilize both pathways indicates IPEC–J2 are more physiologically relevant to study normal intestinal energy metabolism than Caco‐2 cells, which depend solely on glycolysis [31].

However, despite its biological relevance, there is a lack of standardized culture protocols for IPEC–J2 monolayers. Culture conditions, particularly media composition, strongly influence epithelial phenotype and barrier integrity, leading to substantial variability in TEER values across studies. For example, monolayers grown in DMEM/F12 supplemented with 10% fetal bovine serum (FBS) can reach TEER values exceeding 2000–15,000 Ω* × *cm^2^, whereas the use of porcine serum (PS) yields considerably lower values (∼300–500 Ω* × *cm^2^) [22]. Additional variations arise from the use of supplements such as epidermal growth factor (EGF), insulin–transferrin–selenium (ITS) [32, 33], or L‐WRN conditioned medium [34].

Moreover, the passage number is not provided by the culture collection (Leibniz Institute DSMZ‐German Collection of Microorganisms and Cell Cultures), making it difficult to assess how subculturing influences barrier development. As a result, reproducibility remains a major challenge across experiments and between laboratories.

Despite the widespread use of IPEC–J2 for studying microbial interactions, its application to digested food components has been extremely limited, with only one prior report using pea flour digesta [35]. However, the origin of IPEC–J2 from the neonatal piglet makes it particularly suited to infant nutrition studies. In addition, IPEC–J2 cells were harvested from the jejunum, making this cell line highly relevant to dietary protein absorption studies, as free AAs and small peptides are absorbed at this location in the gastrointestinal tract. The present study represents a pioneering effort to characterize IPEC–J2 responses to digested infant formula and to optimize culture conditions for nutrient absorption research in infant nutrition.

The primary objective of this study was to evaluate culture media composition to support IPEC–J2 monolayers with reproducible TEER. Stable IPEC–J2 monolayers could then be assessed for their biocompatibility with digested infant food. We hypothesized that a good medium for IPEC–J2 should sustain a stable and reproducible TEER, maintain metabolic activity and differentiation, preserve an enterocyte‐like phenotype and be sufficiently robust for experiments with digested food.

## 2 Materials and Methods

### 2.1 Materials

The cell line IPEC–J2 (ACC 701) was purchased from the Leibniz Institute DSMZ‐German Collection of Microorganisms and Cell Cultures. Six‐well plates with polyester permeable‐membrane inserts (0.4 µm pore size, 4.5 cm^2^ area), 12‐well plates with polyester permeable‐membrane inserts (0.4 µm pore size, 1.11 cm^2^ area), and 96‐well tissue culture‐treated plates (3300, CellBIND) were purchased from Corning (USA). The Lactate Dehydrogenase Colorimetric Activity Kit (EEA013) was supplied by Invitrogen (MA, USA). CellTiter 96 AQueous One Solution Cell Proliferation Assay (MTS, G3582). RNeasy mini kit (74104) and RNA‐free DNase set (79254) were from Qiagen (Netherlands). SensiFAST cDNA Synthesis Kit (BIO‐65053) was from Meridian Bioscience (UK). Light Cycler SYBR GREEN I Master kit (04707516001) was from Roche Diagnostics (Switzerland). Primers for RT‐qPCR were synthesized by Eurofins Genomics (Germany). All the other reagents were purchased from Sigma‐Aldrich, unless stated otherwise.

In accordance with the principles of the 3Rs (Replacement, Reduction, and Refinement), jejunal mucosal samples were not collected specifically for this study but were obtained from a previously conducted pig study performed at the Teagasc Pig Development Department, Moorepark, Ireland [36]. All procedures were approved by the Teagasc Animal Ethics Committee (TAEC2020‐248) and authorised by the Health Products Regulatory Authority (AE19132/P111), in accordance with EU Directive 2010/63/EU. Briefly, weaned pigs (28 days old) were individually penned and fed twice daily for 28 days. The composition of the diet is provided in Table S1. Pigs had ad libitum access to water. Pigs remained healthy for the duration of the trial. Jejunal mucosal scrapings were collected at slaughter by Dold et al. (2024) [37]. Briefly, jejunal segments were rinsed with sterile PBS, opened longitudinally, and the mucosal layer was gently scraped using a glass slide [37]. Scrapings were snap‐frozen in liquid nitrogen and stored at −80°C until further processing. For the present study, jejunal mucosal scrapings from four pigs (*n* = 4; two males and two females) were randomly and blindly selected.

### 2.2 Cell Culture

#### 2.2.1 Culture Resuscitation and Maintenance

IPEC–J2 cells were maintained in 75 cm^2^ tissue culture flasks at 37°C in a humidified incubator with 5% CO_2_. Routine culture was performed according to the recommendations from the DSMZ cell line collection (ACC 701) in Dulbecco's Modified Eagle Medium/Nutrient Mixture F‐12 (DMEM/F‐12, D8437), supplemented with heat‐inactivated 10% (v/v) FBS (F7524) and 100 U/mL Penicillin‐Streptomycin (P4333), referred to as FBS10. Cells were passaged at 80% confluence every 2–3 days using 0.25% Trypsin‐EDTA solution (T4049). Cells were used at passages 2–5 (with passage 1 defined as the first passage after resuscitating the stock received from the supplier). Cell counting was performed using Trypan blue exclusion and a TC20 automated cell counter (Bio‐Rad, USA).

#### 2.2.2 Experimental Media

For experimental assays, IPEC–J2 cells in FBS10 were seeded onto appropriate supports depending on the experimental condition. After a 16‐h attachment period, cells were washed twice with HBSS, and the culture medium was replaced with one of the following:

1. DMEM/F‐12 + 10% PS + 100 U/mL Penicillin–Streptomycin, referred to as PS10 [22],
2. DMEM/F‐12 + 5% PS + 100 U/mL Penicillin–Streptomycin + 1× Insulin‐Transferrin‐Selenium (ITS, I3146) + 5 ng/mL Epidermal Growth Factor (EGF, E5036), referred to as PS5+EGF+ITS [22],
3. 1:1 (v/v) mixture of PS10 and L‐WRN conditioned medium (SCM105; Sigma–Aldrich), resulting in a final serum concentration of 5%, referred to as PS5 (L‐WRN); or
4. DMEM/F‐12 + 10% FBS + 100 U/mL Penicillin–Streptomycin, referred to as FBS10. Prior to use, PS was heat‐inactivated (60°C for 30 min) and filtered via a 0.45 µm syringe filter (Filtropur S, PES, Sarsted).

### 2.3 TEER

#### 2.3.1 Real‐Time TEER Monitoring

To assess epithelial barrier formation and integrity over time, TEER measurements were performed. IPEC–J2 cells were seeded at a density of 1.2 × 10^5^ cells/mL in 2 mL FBS10 onto polyester permeable membrane inserts (6‐well format; apical chamber: 2 mL, basolateral chamber: 3 mL, according to insert specifications). After 16 h of incubation at 37°C, the cells were washed with HBSS and the medium was replaced with one of the following: PS10, PS5+EGF+ITS, PS5+L‐WRN, or FBS10 (one insert per condition). The inserts were transferred to the CellZscope system (nanoAnalytics GmbH, Germany) for continuous TEER measurements (Ω* × *cm^2^). Following transfer to the CellZscope system, both apical and basolateral chambers were adjusted to 4 mL of the corresponding medium, in accordance with the instrument requirements for stable TEER measurements. The electrical impedance was not altered with different media, so one insert without cells was included as a blank control for TEER background values within each run. TEER was recorded every 24 h for 14 days. Culture medium was replaced every two days, 16 h prior to the TEER measurement. Due to the 6‐well format of the CellZscope system, only one insert per condition could be measured per run, and therefore no technical replicates were included. To ensure robustness, the experiment was independently repeated four times on separate days, with conditions randomly assigned across wells in each run.

#### 2.3.2 Endpoint TEER Measurements

For experiments involving differentiated monolayers, IPEC–J2 cells were seeded at 1.2 × 10^5^ cells/mL in 12‐well Transwell plates, with 0.5 mL FBS10 in the apical chamber and 1.5 mL FBS10 added to the basolateral chamber (according to insert specifications). After a 16 h incubation, cells were washed twice with HBSS, and the medium was replaced with PS10, PS5+EGF+ITS, PS5+L‐WRN, or FBS10 at the same volumes. Media was changed every two days, and 16 h prior to TEER measurements. Cells were cultured for 14 days to allow the formation of a differentiated epithelial barrier, as indicated by stable TEER [38] values and alkaline phosphatase (ALP) activity [39]. TEER was measured at day 7 and day 14 using a Millicell‐ERS Voltohmmeter (Ω* × *cm^2^; Merck Millipore). One insert without cells was included as a blank control. Each culture condition was tested in duplicate in at least three independent experiments.

### 2.4 Alkaline Phosphatase (ALP) and Lactate Dehydrogenase (LDH) Activity

ALP and LDH activity were quantified as indicators of epithelial differentiation and metabolic activity, respectively. IPEC–J2 monolayers were cultured for 7 or 14 days in 12‐well Transwell plates and assessed for ALP and LDH activity. After incubation, cells were washed twice with PBS and lysed in 0.5 mL of 0.2% Triton X‐100 (prepared in Milli‐Q H_2_O) by shaking at room temperature for 20 min. Total protein concentration in cell lysates was determined using the bicinchoninic acid (BCA) assay (Pierce, Thermo Fisher Scientific) according to the manufacturer's instructions, and enzyme activities were normalized to total protein content. Each condition was tested in technical duplicate across at least three independent experiments.

#### 2.4.1 ALP Activity Assay

For ALP activity, 50 µL of cell lysate was transferred to a clear, flat‐bottom 96‐well plate. The assay was performed using the ALP Assay Kit (MAK447), which is based on the enzymatic conversion of p‐nitrophenyl phosphate into p‐nitrophenol and inorganic phosphate. Absorbance was measured at 405 nm using a Synergy HT plate reader (Supplier, Country) at timepoints 0 and 4 min. A reagent blank was included, and its absorbance was subtracted to correct for background color.

ALP activity was calculated using the following formula:

$$\frac{\left[\left(OD_{T4}-OD_{T0}\right)\times \mathrm{RxnVol}\right]}{\varepsilon \mathrm{PNP}\times L\times \mathrm{SmplVol}\times t}$$

where, OD_T0_ = absorbance at 405 nm at time 0, OD_T4_ = absorbance at 405 nm at time 4 min; RxnVol = reaction volume; εPNP = molar absorptivity of p‐nitrophenol (18.75 mM^−^^1^ cm^−^^1^); *L* = path length in cm; SmplVol = sample volume; *t* = reaction time (4 min).

The optical path length (L) was calculated as:

$$L=\left(OD_{\mathrm{Cal}}-OD_{\mathrm{Blank}}\right)/\left(\varepsilon C\right)$$

where, OD_Cal_ = absorbance at 405 nm of the kit calibrator; OD_Blank_ = absorbance at 405 nm of the blank (medium only); ε = molar absorptivity; C = path length of the plate

#### 2.4.2 LDH Activity Assay

For LDH activity, 5 µL of cell lysate was transferred to a clear, flat‐bottom 96‐well plate. The assay was performed using the LDH Colorimetric Activity Kit, which measures the conversion of lactate to pyruvate. Pyruvate reacts with 2,4‐dinitrophenylhydrazine to form a colored pyruvate‐hydrazone product. Absorbance at 450 nm was recorded at 3 and 6 min using a Synergy HT plate reader (Supplier, Country). Absorbance values were corrected for any background absorbance from reagents. LDH activity was calculated as:

$$\mathrm{LDH}\;\mathrm{activity}=\frac{B}{\left(T\times V\right)\times \mathrm{Sample}\;\mathrm{Dilution}\;\mathrm{Factor}\left(20\right)}$$

where, B = nmoles of NADH generated between timepoints T_3_ and T_6_, calculated from a standard curve; T = reaction time (3 min); V = sample volume (5 µL); dilution factor = 20.

### 2.5 Lactulose: Mannitol Permeability Assay

To assess permeability across the epithelial monolayer, lactulose–mannitol transport was measured. The protocol for assessing paracellular permeability using lactulose and mannitol was adapted from Kondrashina et al. (2021). Lactulose (25 mg/mL, 61360‐25G) and mannitol (5 mg/mL, M9546‐250G) were dissolved in HBSS, mixed in a 1:1 volume ratio (1 part lactulose solution to 1 part mannitol solution) to generate a 2.5× working stock, and filter‐sterilized using 0.2 µm filters. IPEC–J2 monolayers were cultured for 14 days in 12‐well Transwell inserts and washed twice with HBSS. Monolayers were equilibrated at 37°C for 30 min in HBSS (apical: 0.3 mL; basolateral: 1.5 mL). After equilibration, 200 µL of the lactulose mannitol solution was added to the apical chamber, and cells were incubated for 4 h at 37°C. TEER was recorded at time 0 and after 4 h to monitor barrier integrity. Apical and basolateral samples were then collected and stored at −20°C for analysis. Lactulose and mannitol concentrations were measured using high‐performance anion‐exchange chromatography with pulsed amperometric detection (HPAEC‐PAD) on an ICS‐3000 system (Dionex, USA) equipped with an electrochemical detector. Apical samples were diluted 1:99 in Milli‐Q water, while basolateral samples were diluted 1:1. All samples were filtered through a 0.2 µm nylon syringe filter prior to injection.

Separation was performed on a CarboPac PA100 analytical column (4 × 250 mm) preceded by a PA100 guard column (4 × 50 mm), with a flow rate of 1 mL/min. The mobile phases consisted of: Eluent A = 100 mM NaOH; Eluent B = 100 mM NaOH+500 mM sodium acetate (NaOAc). The elution gradient was as follows: from t = 0 to 6 min, 100% Eluent A; at t = 13 min, 51.4% Eluent A and 48.6% Eluent B; from t = 13.1 to 23.1 min, equilibrated at 100% Eluent A.

Lactulose and mannitol levels were quantified by comparing peak areas with known standards (10 mg/L). The results were expressed as lactulose and mannitol apparent permeability (Papp) calculated with the following formula:

$$\mathrm{Papp}\left(\mathrm{cm}/\min\right)=\frac{\left(C_{\mathrm{Baso}}\times V\right)}{\left(t\times A\times C_{\mathrm{In}}\right)}$$

where, C_Baso_ = concentration of lactulose or mannitol in the basolateral compartment (mg/L); *V* = volume of the basolateral compartment (L); *t* = incubation time (14400 sec); *A* = surface area (1.1 cm^2^); C_In_ = initial concentration in the apical (mg/L).

The lactulose:mannitol ratio was also calculated in the 1X stock solution and in both apical and basolateral compartments after 4 h of incubation to evaluate changes in selective permeability. The osmotic contribution of the lactulose:mannitol solution was ∼20 mOsm/L, estimated based on solute concentrations and molecular weights using the van't Hoff equation:

$$\begin{array}{cc} & \mathrm{Osmolarity}\left(\frac{\mathrm{mOsm}}{L}\right)\\ & \;=\left(\mathrm{Concentration}\left(\frac{G}{L}\right)/\mathrm{Molecular}\mathrm{Weight}\left(\frac{G}{\mathrm{Mol}}\right)\right)\times I\times 1000 \end{array}$$

The experiment was performed in biological triplicate with technical duplicates.

### 2.6 Reverse Transcription Quantitative PCR (RT‐qPCR)

To evaluate mRNA transcript levels of markers of epithelial function and differentiation, RT‐qPCR analysis was performed. IPEC–J2 monolayers cultured for 14 days, in 12‐well Transwell inserts with the various test media (*n* = 3 independent biological replicates), were washed twice with PBS and lysed by adding 350 µL of RLT buffer (Qiagen). After 5 min on ice, the lysates were collected by pipetting and stored at −80°C.

For both the cell lysates and the porcine jejunal mucosal scrapings, RNA extraction was performed using the RNeasy Mini Kit (Qiagen), including an on‐column DNase digestion step, according to the manufacturer's instructions. Total RNA concentration and purity were assessed using a NanoDrop 1000 spectrophotometer (Thermo Fisher Scientific, MA, USA). Only samples with an OD_260_/_280_ ratio > 1.8 were used for downstream analysis.

Complementary DNA (cDNA) was synthesized from 0.5 µg (IPEC–J2 samples) or 700 ng (mucosal scrapings) of total RNA using the SensiFAST cDNA Synthesis Kit (Bioline). Quantitative PCR was carried out in technical duplicates using the LightCycler SYBR Green I Master Kit (Roche) on a LightCycler 96 instrument. Primer sequences are listed in Table S2. Where necessary, primers were designed using Primer‐BLAST (Ye et al., 2012) based on porcine gene sequences from GenBank. The RPLP0 gene, encoding ribosomal protein lateral stalk subunit P0, was used as the housekeeping reference. Transcript levels were normalized to the FBS10 condition, which represents the standard medium supplied with the cell line collection. Amplification efficiency ranged between 90%–110%, and the optimal annealing temperature was 60°C. Relative gene expression was calculated using the ΔΔCt method:

$$\begin{array}{cc} \Delta \Delta \mathrm{Ct} & ={\left(\mathrm{Ct}\;\mathrm{Target}\;\mathrm{gene}-\mathrm{Ct}\;RPLP0\right)}_{\mathrm{test}}\\ & \;-{\left(\mathrm{Ct}\;\mathrm{Target}\;\mathrm{gene}-\mathrm{Ct}\;RPLP0\right)}_{\mathrm{Control}} \end{array}$$

where, Ct = threshold cycle for either the target gene or RPLP0; test refers to any experimental condition, control refers to FBS10 monolayers. Relative expression levels are presented as fold changes (2*^−^* ^ΔΔCt^), while statistical analyses were performed on the ΔΔCt values.

### 2.7 Immunofluorescence Staining of Zonula Occludens‐1 (ZO1)

To assess tight junction organization and localization, ZO1 immunofluorescence staining was performed. IPEC–J2 monolayers cultured for 14 days in PS10 or PS5+EGF+ITS were fixed directly in Transwell inserts. Fixation was performed by adding 1 mL of ice‐cold methanol to both the apical and basolateral chambers for 10 min. Following fixation, cells were washed twice with PBS (1 mL per chamber) using a transfer pipette. Permeabilization was performed for 5 min at room temperature with 0.5% Triton X‐100 in PBS (1 mL per chamber), followed by two additional PBS washes. Monolayers were then incubated for 1.5 h at 37°C, on the apical side, with 100 µL ZO1 primary antibody directly conjugated to Alexa Fluor 594 (Ref. 3391914, Lot 1364021A; 1:99 dilution in 0.2% BSA in PBS), enabling direct fluorescence detection without the use of a secondary antibody. Inserts were then washed twice with PBS and rinsed briefly in Milli‐Q water. The membrane inserts were carefully excised from the Transwell supports and mounted onto microscope glass slides using 15 µL of ProLong Gold Antifade Mountant with DAPI (Thermo Fisher Scientific, P36931). Coverslips were applied and sealed. Fluorescence imaging was performed using a Zeiss Axio Observer equipped with an ApoTome.2 structured illumination system and a Plan‐Apochromat 40×/1.3 Oil objective. Images were acquired using fixed exposure times: DAPI (50 ms, blue channel) and Alexa Fluor 594 (100 ms, red channel). Image processing and analysis were performed using Fiji/ImageJ software. For each field of view, a region of interest (ROI) was manually selected to include areas with well‐defined nuclei. The Otsu thresholding algorithm was applied to the ZO1 channel to distinguish signal from background, and the integrated fluorescence intensity within the ROI was measured. ZO1 intensity was then normalized to the number of nuclei within the same ROI. Nuclei were counted from the DAPI channel and nuclear density was expressed as the number of nuclei per 1000 µm^2^. Images were acquired from monolayers derived from three independent biological replicates.

### 2.8 Preparation and Characterization of Dairy Infant Formula (IF) Digesta

#### 2.8.1 Simulated Gastro‐Intestinal Digestion

Simulated gastrointestinal digestion was performed to mimic physiological digestion and produce bioaccessible fractions to treat IPEC–JC monolayers. A dairy‐based IF was reconstituted according to the manufacturer's instructions by dissolving 4.3 g of powder in 30 mL of Milli‐Q water. Simulated infant gastrointestinal digestion was performed in triplicate based on the protocol by Ménard et al. (2018), with modifications. For each digestion, 5 mL of reconstituted Dairy IF or Milli‐Q water (negative control) was mixed with 2.94 mL of infant gastric simulated fluid containing 268 U/mL pepsin (RGE15, pepsin activity: 596 U/mg measured in‐house). The pH was adjusted to 5.3 with 1 M HCl, and samples were incubated at 37°C for 1 h in a rotating wheel (100 rpm). Following the gastric phase, the pH was raised to 7.0 using 1 M NaOH to inactivate pepsin. Next, 4.88 mL of simulated intestinal fluid was added, containing 16 U/mL pancreatin (trypsin activity: 8.9 U/mg measured in‐house) and 3.1 mM bovine bile salts. The pH was adjusted to 6.6 with 1 M HCl, and samples were incubated again at 37°C for 1 h in the rotating wheel. After the intestinal phase, digesta were inactivated by either (i) heating at 95°C for 10 min (HI), or (ii) addition of 4‐(2‐aminoethyl)benzenesulfonic fluoride hydrochloride (Pefabloc; final concentration: 1 mM) and Orlistat (final concentration: 0.1 mM in ethanol) (P+O). Reconstituted dairy IF with the addition of gastric enzymes, followed by immediate inactivation with heat at 95°C for 10 min represented dairy IF before digestion (dairy IF‐G0). A water control was included where H_2_O replaced dairy IF in the digestion (H_2_O G0, H_2_O (HI) and H_2_O (P+O). All digestions were performed in triplicate on separate days. Digested samples were aliquoted and stored at −20°C until further analysis.

#### 2.8.2 SDS Page

To provide evidence of digestion, digested samples were analyzed by SDS‐PAGE, and degree of protein hydrolysis measured. Protein concentration of dairy IF G0, dairy IF (HI), and dairy IF (P+O) was determined using the bicinchoninic acid (BCA) assay (Pierce, Thermo Fisher Scientific, Ireland), according to the manufacturer's instructions. For each sample, 10 µg protein was prepared under reducing conditions in loading buffer and loaded onto a NuPAGE 10% Bis‐Tris gel (Thermo Fisher Scientific) with MOPS running buffer. Samples were heated at 95°C for 5 min prior to loading. Electrophoresis was performed using an XCell SureLock Mini‐Cell system at 150 V for 60 min.

A pre‐stained protein ladder (PageRuler, 10–180 kDa, Thermo Fisher Scientific) was used as a molecular weight marker. After electrophoresis, gels were stained overnight with InstantBlue Protein Stain (Expedeon) and destained in Milli‐Q water.

#### 2.8.3 Degree of Protein Hydrolysis—O‐Phthaldialdehyde (OPA) Assay

The extent of protein hydrolysis was also assessed by measuring free amino groups using the o‐phthaldialdehyde (OPA) assay. Dairy IF‐G0, dairy IF (HI), and dairy IF (P+O) samples were thawed on ice and precipitated with 5% trichloroacetic acid (TCA) by mixing 0.5 mL of sample with 0.83 mL of TCA. After centrifugation at 1000 g for 30 min, the supernatants were collected and diluted 1:1 (dairy IF G0) or 1:4 (dairy IF (HI)/ P+O) in 0.1 M borate buffer. The OPA reagent was freshly prepared using 0.1 M borate, 10% SDS, 40 g/L OPA in ethanol, and 200 g/L N‐acetyl‐L‐methionine ethyl ester. For each sample, 8 µL was added to 232 µL of OPA reagent in a UV 96‐well plate, followed by incubation in the dark at 30°C for 10 min. Absorbance was measured at 340 nm using a PowerWave XS microplate reader (BioTek Instruments, USA). Quantification was based on a glutamic acid standard curve (0–8 mmol/L). All measurements were performed using digesta generated in three independent experiments (biological replicates), with each sample analyzed in technical duplicate. The concentration of free NH_2_ groups (mmol) was normalized to protein content (g), as determined by the BCA assay.

### 2.9 IPEC–J2 Exposure to Digesta

#### 2.9.1 MTS Assay

To assess the cytotoxicity of digested dairy IF samples, IPEC–J2 cells were seeded in 96‐well tissue culture‐treated plates at a density of 1 × 10^5^ cells/mL in either PS10 or PS5+EGF+ITS medium (150 µL per well) and incubated for 16 h at 37°C in 5% CO_2_.

Dairy IF (HI) and dairy IF (P+O) were thawed at room temperature, diluted in HBSS to final concentrations corresponding to 400, 350, 300, 250, 200, 150, and 100 µg protein/cm^2^, and filter‐sterilized using 0.2 µm syringe filters. Media was removed from overnight cultures. IPEC–J2 cells were then washed with HBSS. Diluted dairy IF digested samples (150 µL) were then added to the cells and incubated for 2 h at 37°C. After exposure, the digesta was removed, cells were gently washed with HBSS, and cell viability was assessed using the CellTiter 96 AQueous One Solution Cell Proliferation Assay (Promega).

Briefly, 100 µL of MTS reagent, diluted 1:10 in HBSS, was added to each well, and cells were incubated for 1 h at 37°C. Absorbance was then measured at 490 nm using a BioTek Cytation 5 multimode plate reader. Cell viability was expressed as a percentage relative to control wells treated with HBSS alone. Cells in HBSS buffer were 84.26% ± 6.10% viability of cells in PS10 or PS5+EGF+ITS alone. The assay was performed on three different days using digesta generated in three independent experiments, with each condition tested in technical duplicate.

#### 2.9.2 Transwell Exposure

To evaluate the impact of dairy IF digesta on epithelial barrier integrity and nutrient bioavailability, differentiated IPEC–J2 monolayers were exposed to the digesta. IPEC–J2 cells cultured for 14 days on 12‐well Transwell inserts were washed twice with HBSS and incubated in HBSS for 30 min at 37°C to allow acclimatization (0.5 mL in the apical and 1.5 mL in the basolateral compartments). Digested dairy IF samples (HI and P+O) were thawed at room temperature, diluted in HBSS to final concentrations of 550 µg protein/mL and 330 µg protein/mL, respectively. Samples were then filtered through low protein‐binding 0.45 µm PES syringe filters (Sarstedt) and pre‐warmed to 37°C. Next, 0.5 mL of each sample or HBSS (untreated control) was applied to the apical compartment, resulting in final surface loading concentrations of 250 µg protein/cm^2^ for dairy IF (HI) and 150 µg protein/cm^2^ for dairy IF (P+O). These concentrations were selected based on preliminary MTS viability assays, which identified the highest concentrations that did not adversely affect cell viability for each treatment. H_2_O (HI) and (P+O) were included as negative controls. TEER was measured immediately after acclimatization (T0) and again after 2 h of treatment with digesta samples (T2). Apical and basolateral samples were then collected, aliquoted, and stored at −20°C for subsequent AA analysis. Digesta generated from three independent experiments (biological replicates) were tested, with technical duplicates included on each experimental day.

#### 2.9.3 Free AA Analysis

Free AA analysis was performed in both apical and basolateral chambers post‐IF treatment, as an indicator of AA transport across epithelial monolayers. Two technical replicates were pooled from the apical and basolateral chambers. Basolateral samples were concentrated using a vacuum concentrator (miVac quattro concentrator, Genevac) at 40°C and resuspended in 600 µL of Milli‐Q water. For comparison purposes, dairy IF (HI) and (P+O) samples were diluted 1:9 (v/v) in Milli‐Q water and included in the analysis. Samples were deproteinized by mixing equal volumes of 24% (w/v) trichloroacetic acid (TCA) with sample, followed by incubation at room temperature for 10 min. The mixtures were then centrifuged at 14,400 × g for 10 min (Microcentaur, MSE, UK). Supernatants were collected and diluted 1:15 with 0.2 M sodium citrate buffer (pH 2.2) containing the internal standard norleucine at a final concentration of 125 nmol/mL. Quantification of free AAs was performed using a Jeol JLC‐500/V AA analyzer (Jeol (UK) Ltd., Garden City, Herts, UK) equipped with a Jeol Na^+^ high‐performance cation exchange column. AA concentrations were expressed in nmol/mL. The basolateral AA concentration was expressed as a ratio of the apical concentration using the following formula:

$$\mathrm{Ratio}=\frac{\left(\mathrm{basolateral}\;\mathrm{concentration}\;\mathrm{at}\;2\;h\right)}{\left(\mathrm{apical}\;\mathrm{concentration}\;\mathrm{at}\;\mathrm{time}\;0\right)}$$

### 2.10 Statistical Analysis

All experiments were performed using at least three independent biological replicates conducted on separate days, each with technical duplicates, unless otherwise stated. Data are presented as mean ± SEM. Statistical analyses were performed using GraphPad Prism (version 10.4.2; GraphPad Software, CA, USA). Data distribution was assessed for normality using the Shapiro–Wilk test. All tests were two‐tailed, and *p* < 0.05 was considered statistically significant.

Depending on the experimental design, differences between groups were analysed using one‐way or two‐way ANOVA, including repeated measures ANOVA for time‐course data, followed by Tukey's multiple comparisons test. Where comparisons were made against a single control group, Dunnett's test was applied. Pairwise comparisons were performed using Student's t‐tests. For analyses involving multiple comparisons across related variables, P‐values were adjusted using the Benjamini–Hochberg false discovery rate (FDR) method.

Assay‐specific applications included the use of two‐way repeated measures ANOVA for TEER time‐course experiments, two‐way ANOVA with Dunnett's test for viability assays (factors: concentration and treatment), and one‐way ANOVA or Student's t‐tests for endpoint measurements, enzyme activity assays, permeability assays, and imaging‐based analyses, as appropriate. RT‐PCR data are presented as geometric mean (2*^−^* ^ΔΔCt^) with 95% confidence intervals. Statistical analyses were performed on log_2_‐transformed ΔΔCt values using Student's t‐tests or, for multiple group comparisons, one‐way ANOVA with Tukey's test or Kruskal–Wallis with Dunn's test, as appropriate. For TEER measurements following digesta exposure, analyses were conducted separately for each medium, with treatment effects assessed by one‐way ANOVA and timepoint comparisons by paired Student's t‐tests. AA transport ratios were analysed using two‐way ANOVA with medium and digesta condition as factors, while other AA comparisons were performed using Student's t‐tests with FDR correction.

In graphical representations, different letters indicate statistically significant differences between groups within the same condition, while asterisks denote significant pairwise differences.

## 3 Results

Four media conditions (PS10, PS5+EGF+ITS, PS5+L‐WRN, and FBS10) were evaluated for their ability to support IPEC–J2 monolayer formation and barrier function.

### 3.1 Media Dictates TEER Values of IPEC–J2 Monolayers

**FIGURE 1:**
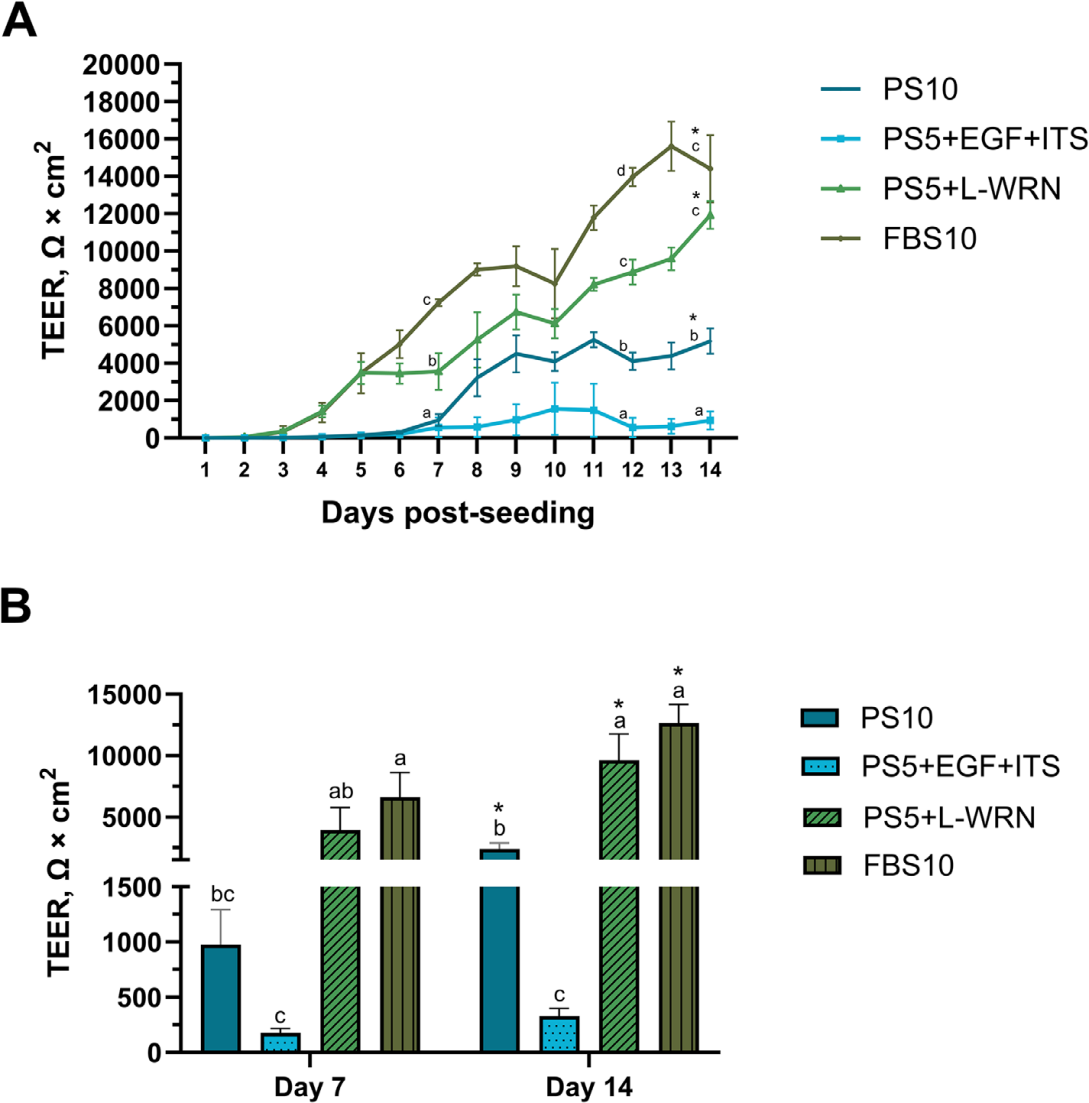
Transepithelial electrical resistance (TEER) measurements in IPEC–J2 monolayers cultured under different media conditions. (A) TEER values monitored over time using the real‐time CellZScope system. (B) Endpoint TEER values measured at selected timepoints using the Millicell‐ERS voltameter. Data represent mean ± SEM from three biological replicates. Real‐time measurements (A) were obtained from single inserts, while endpoint measurements (B) were performed in technical duplicates. Statistical analysis for panel (A) was performed using two‐way repeated measures ANOVA with Tukey's post hoc test. For panel (B), differences between media were analyzed using one‐way ANOVA with Tukey's post hoc test, and differences between day 7 and day 14 within each condition were assessed using unpaired Student's t‐tests. Different letters indicate statistically significant differences between conditions at the same time point; asterisks denote significant differences between day 7 and day 14 within the same condition. (*p < *0.05).

IPEC–J2 cells were cultured for 14 days on permeable Transwell inserts in PS10, PS5+EGF+ITS, PS10+L‐WRN or FBS10 media. As a first step in media assessment, barrier integrity of monolayers was assessed by TEER in real‐time (CellZScope) and by endpoint (Millicell) (Figure 1A,B). Using the CellZScope system, monolayer TEER values at day 7 were: PS10 = 963.11 ± 318.66, PS5+EGF+ITS = 566.11 ± 528.82, PS5+L‐WRN =  3560.51 ± 979.54, and FBS10 = 7241.50 ± 186.85 Ω × cm^2^ (Figure 1A). At this time point, values for monolayers in PS10 and PS5+EGF+ITS were not significantly different from each other (*p* > 0.05), whilst culturing in PS5+L‐WRN was significantly higher than both PS10 and PS5+EGF+ITS (*p* < 0.05), and culturing in FBS10 was significantly higher again (*p* < 0.05). By day 14, IPEC‐J2 TEER had increased with 3 of the media: PS10 = 5178.61 ± 675.74, PS5+L‐WRN = 11,931.56 ± 740.28, and FBS10 = 14,412.56 ± 1798.99 Ω × cm^2^. However, monolayer TEER values cultured in PS5+EGF+ITS was not significantly different to day 7 (943.13 ± 487.39 Ω × cm^2^; *p* > 0.05). At this stage, IPEC–J2 TEER values for PS5+L‐WRN and FBS10 were similar (*p* > 0.05) and significantly higher than for PS10 and PS5 +EGF/ITS (*p* < 0.05). Notably, monolayers cultured in PS10 had significantly higher TEER values than monolayers in PS5+EGF+ITS (*p* > 0.05). Importantly, TEER values at day 12 were not significantly different from those at day 14 within each condition (*p* > 0.05), indicating that the epithelial barrier had stabilized by day 12 (Figure 1A).

Using the Millicell‐ERS system revealed IPEC–J2 TEER values at day 7 of 978.08 ± 314.45 cultured in PS10, 176.53 ± 40.81 in PS5+EGF+ITS, 3937.52 ± 485.51 in PS5+L‐WRN, and 6620.78 ± 1997.03 Ω × cm^2^ in FBS10 (Figure 1B). By day 14, TEER values further increased to: PS10 = 2395.00 ± 485.51, PS5+L‐WRN = 9600.00 ± 2150.94, and FBS10 = 12,660.27 ± 1495.01 Ω × cm^2^. In contrast, IPEC–J2 cultured in PS5+EGF+ITS displayed significantly lower TEER values (327.58 ± 71.58 Ω × cm^2^; *p* < 0.05) compared with all other treatments, and these did not increase significantly from day 7 to day 14. Among the other conditions, TEER values were highest in FBS10 and PS5+L‐WRN, with no significant difference between the two (*p* > 0.05). Monolayers cultured in PS10 also exhibited significantly higher TEER values than those in PS5+EGF+ITS (*p* < 0.05).

### 3.2 ALP and LDH Activity Reflect Media‐Dependent IPEC–J2 Differentiation

**FIGURE 2:**
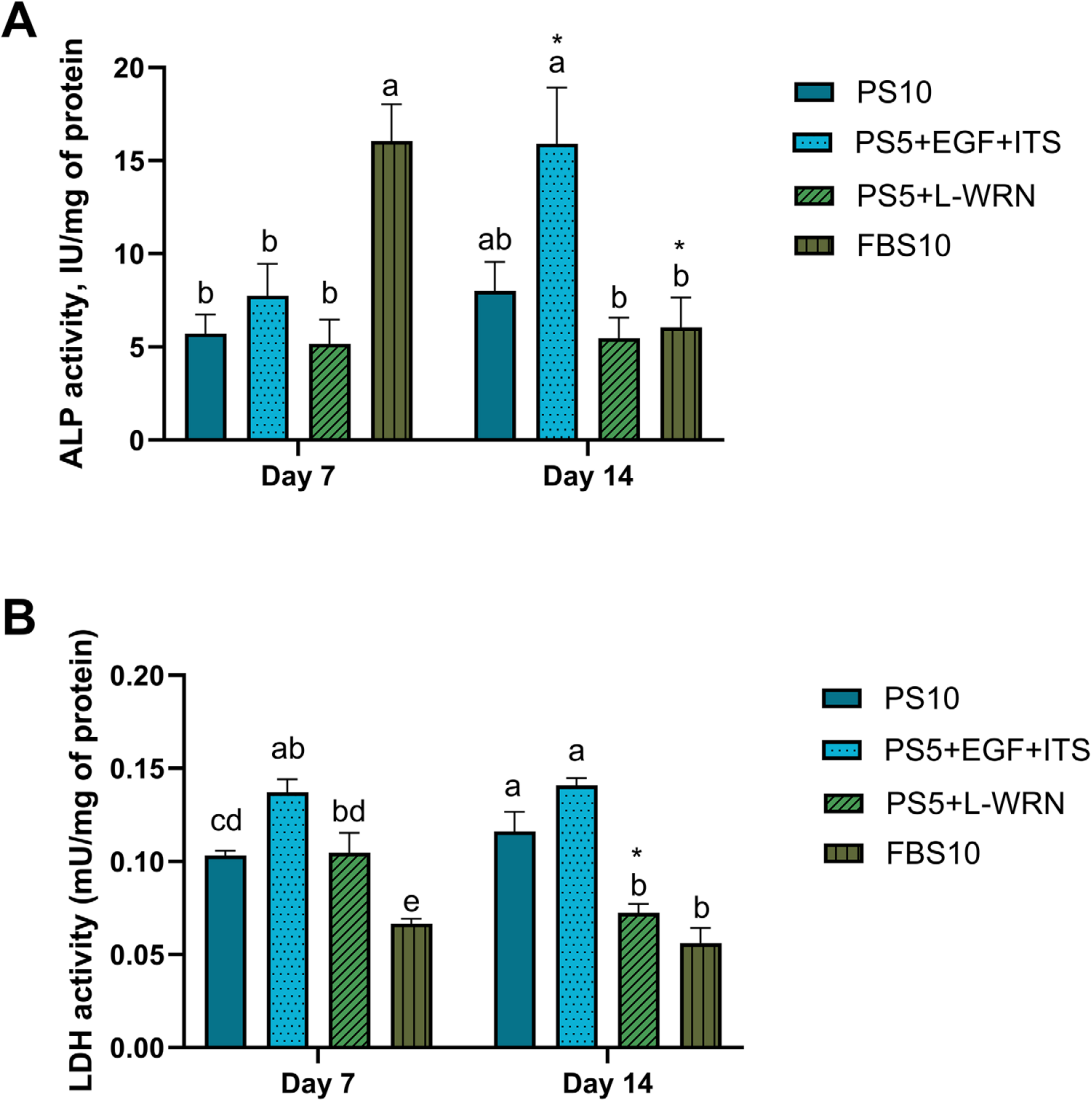
Alkaline phosphatase (ALP) and lactase dehydrogenase (LDH) activity in IPEC–J2 monolayers at days 7 and 14. IPEC–J2 cells were cultured in PS5+EGF+ITS, PS10, PS5+L‐WRN, or FBS10 media for 14 days on permeable Transwell inserts. (A) ALP activity and (B) LDH activity were measured in cell lysates on days 7 and 14. Activities were expressed per cellular protein content. Data represent mean ± SEM from three biological replicates and technical duplicates. Statistical analysis was performed using one‐way ANOVA followed by Tukey's post hoc test to assess differences between treatments at the same time‐point. Differences between day 7 and day 14 within each condition were assessed using unpaired Student's t‐tests. Asterisks indicate statistically significant differences between day 7 and day 14 within the same condition, determined by t‐test (*p < *0.05).

To evaluate enterocyte differentiation and metabolic activity in IPEC–J2 monolayers cultured in the various media, ALP and LDH activity were measured on days 7 and 14 (Figure 2). ALP is a well‐known marker of epithelial differentiation [39], and LDH is a key enzyme in anaerobic glycolysis. On day 7, FBS10 resulted in the highest (*p* < 0.05) ALP activity in IPEC–J2 monolayers (FBS10 = 16.06 ± 1.9 IU/mg of protein; PS10 = 5.72 ± 1.02 IU/mg of protein, PS5+EGF+ITS = 7.75 ± 1.72 IU/mg of protein, PS5+L‐WRN = 5.17 ± 1.31 IU/mg of protein; Figure 2A). By day 14, ALP activity significantly increased in monolayers cultured in PS5+EGF+ITS (15.92 ± 3.02 IU/mg of protein; *p* < 0.05 vs. day 7). ALP levels in monolayers cultured in PS10 (8.01 ± 1.54 IU/mg of protein) were not significantly different from those in PS5+EGF+ITS (*p* > 0.05), whereas activity in monolayers with PS5+L‐WRN (5.47 ± 1.10 IU/mg of protein) or FBS10 (6.06 ± 1.60 IU/mg of protein) were significantly lower than monolayers cultured in PS5+EGF+ITS (*p* < 0.05) (Figure 2A). Notably, monolayers cultured in FBS10 exhibited a significant decrease in ALP activity from day 7 to day 14 (*p* < 0.05).

LDH activity showed a similar media‐dependent pattern (Figure 2B). On day 7, the highest activity was observed in monolayers cultured in PS5+EGF+ITS (0.14 ± 0.007 mU/mg of protein; *p* < 0.05), followed by PS5+L‐WRN (0.10 ± 0.01 mU/mg of protein) and PS10 (0.10 ± 0.003 mU/mg of protein), which were both significantly higher than monolayers cultured in FBS10 (0.07 ± 0.003 mU/mg of protein; *p* < 0.05). By day 14, LDH activity remained highest in monolayers in PS5+EGF+ITS (0.14 ± 0.004 mU/mg of protein) and PS10 (0.12 ± 0.01 mU/mg of protein; *p* < 0.05). In contrast, LDH activity was significantly lower in monolayers cultured in PS5+L‐WRN (0.07 ± 0.005 mU/ mg of protein; *p* < 0.05 vs. day 7). LDH activity in IPEC–J2 monolayers cultured in FBS10 remained low at day 14 with values similar to day 7 (0.06 ± 0.008 mU/mg of protein; *p* > 0.05).

### 3.3 PS5+EGF+ITS Increases Lactulose and Mannitol Permeability in IPEC–J2 Monolayers

**FIGURE 3:**
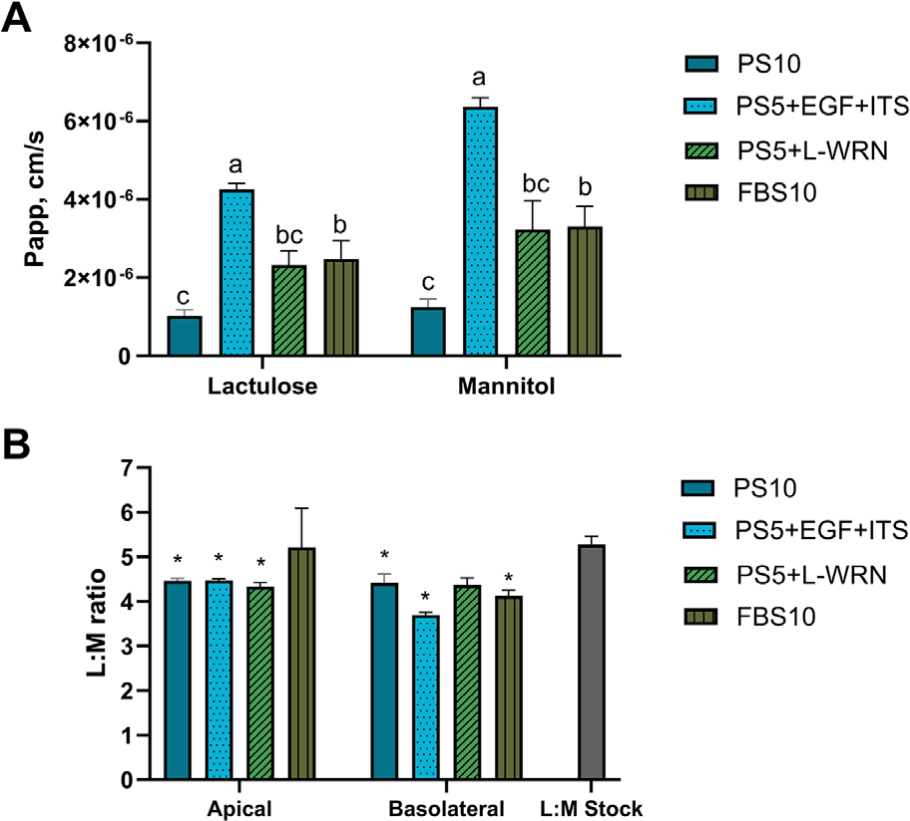
Media‐dependent permeability to lactulose and mannitol in IPEC–J2 monolayers. IPEC–J2 cells were cultured for 14 days in PS10, PS5+EGF+ITS, PS5+L‐WRN, or FBS10 media on permeable Transwell inserts. (A) Apparent permeability coefficients (Papp) of lactulose and mannitol in HBSS were calculated after a 4 h incubation. (B) Lactulose:mannitol (L:M) ratios were determined in the apical and basolateral compartments at 4 h. L:M stock ratio (1:5.27 ± 0.19) = initial input solution applied to the apical side at time 0. Data represent mean ± SEM from three biological replicates and technical duplicates. Statistical analysis was performed using one‐way ANOVA followed by Tukey's post hoc test, with different letters indicating statistically significant differences (*p* < 0.05) between treatments within the same sugar. Student's t‐tests were employed to compare treatment groups to the L:M stock ratio, with asterisks denoting significant differences from the L:M stock.

To assess the permeability of monolayers at day 14, lactulose (radius = 9.5 Å) and mannitol (radius = 6.7 Å) were simultaneously added to apical chambers. After 4 h, levels were quantified in basolateral chambers (Figure 3). Papp of lactulose in IPEC–J2 monolayers revealed that culturing in PS10 resulted in the lowest Papp value (9.66 × 10*^−^* ^7^ ± 1.97 × 10*^−^* ^7^ cm/s), significantly lower than culturing in PS5+EGF+ITS (4.26 × 10*^−^* ^6^ ± 1.53* × *10*^−^* ^7^ cm/s; *p* < 0.05), and FBS10 (2.47 × 10*^−^* ^6^ ± 4.69 × 10*^−^* ^7^ cm/s; *p* < 0.05), albeit similar to PS5+L‐WRN (2.32 × 10*^−^* ^6^ ± 3.70 × 10*^−^* ^7^ cm/s; *p* > 0.05) (Figure 3a).

The Papp for mannitol in monolayers was significantly lower in PS10 (1.15 × 10*^−^* ^6^ ± 2.75 × 10*^−^* ^7^ cm/s) compared to monolayers cultured in either PS5+EGF+ITS and FBS10. PS5+EGF+ITS resulted in the highest mannitol permeability (6.37 × 10*^−^* ^6^ ± 2.33 × 10*^−^* ^7^ cm/s; *p* < 0.05 vs. all others), while culturing in PS5+L‐WRN (3.23 × 10*^−^* ^6^ ± 7.41 × 10*^−^* ^7^ cm/s) and FBS10 (3.31 × 10*^−^* ^6^ ± 5.19 × 10*^−^* ^7^ cm/s) were not significantly different from each other (*p* > 0.05) (Figure 3A)

The lactulose: mannitol ratio of the stock solution applied apically at T_0_ was 1:5.27 ± 0.19 (Figure 3B). This served as a reference point, with lower ratios indicative of preferential mannitol (transcellular) transport, and higher ratios suggestive of increased lactulose (paracellular) transport. In the apical compartment after 4 h, lactulose: mannitol ratios were significantly lower than the stock solution in monolayers cultured in PS10 (1:4.46 ± 0.056), PS5+EGF+ITS (1:4.47 ± 0.038) and PS5+L‐WRN (1:4.33 ± 0.083; *p < *0.05 for all vs. stock), indicating preferential mannitol uptake over lactulose. Lactulose: mannitol ratio in monolayers cultured in FBS10 (5.22 ± 0.087) was not significantly different from the stock (p > 0.05) (Figure 3,B). In the basolateral compartment, the lactulose: mannitol ratio was 1:4.41 ± 0.20 in monolayers cultured in PS10, 1:3.69 ± 0.064 with PS5+EGF+ITS, 1:4.13 ± 0.12; with FBS10), and 1:4.36 ± 0.16 with PS5+L‐WRN. Notably, only the lactulose:manntiol ratio in basolateral chambers of monolayers cultured in FBS10 was similar to lactulose:mannitol ratio in the stock solution (*p* > 0.05; Figure 3,B).

Interestingly, a decrease in monolayer TEER values was observed during the pre‐incubation step with HBSS incubation steps. This TEER reduction was exacerbated by the addition of lactulose mannitol solution, where monolayers were cultured in FBS10 (93.19 ± 5.39%) and PS5+L‐WRN (87.78% ± 0.56%), indicating further loss of barrier resistance. In contrast, the loss in TEER was mitigated by culturing monolayers in PS10 (51.81% ± 24.84%) and PS5+EGF+ITS (23.27% ± 19.2%), indicating these monolayers were more robust. It is important to note that osmotic stress is unlikely to account for the observed TEER reduction, as the estimated increase in osmolality with the addition of lactulose mannitol solution was ∼20 mOsm/L to HBSS (270–305 mOsm/kg) [40].

### 3.4 Media Modulates mRNA Levels of Transport, Barrier, and Immune Genes in IPEC–J2 Monolayers

To investigate the mRNA transcript levels of genes involved in nutrient transport, epithelial structure, and cell type identity, RT‐qPCR was performed on IPEC–J2 monolayers cultured for 14 days in various media (Table 1). To provide an in vivo reference, RT‐qPCR was also performed in jejunal mucosal scrapings from young pigs. IPEC‐J2 mRNA levels were normalized to the housekeeping gene RPLP0 and analyzed using the ΔΔCt method, with FBS10 assigned the reference condition as it is the medium recommended by the supplier. Table 1 shows the relative fold changes (2^–ΔΔCt^) of IPEC–J2 cells under the different culture media conditions, while Table S3 presents the corresponding mRNA levels present in porcine jejunal mucosal scrapings of young pigs.

**TABLE 1:** Relative mRNA expression in IPEC–J2 monolayers cultured under different media conditions. IPEC–J2 cells were cultured on Transwell inserts for 14 days in four media (PS10, PS5+EGF+ITS, PS5+L‐WRN, and FBS10). Messeneger RNA was quantified by RT–QPCR, normalized to the housekeeping gene *RPLP0*, and expressed relative to FBS10 (supplier recommended medium) using the 2^−ΔΔCt^ method (FBS10 = 1). Data are presented as 2^−ΔΔCt^ values ± SEM. Statistical analyses were performed on ΔΔCt values using one‐way ANOVA with Tukey's post hoc test or Kruskal–Wallis with Dunn's multiple comparisons test when normality assumptions were not met. Different superscript letters within the same gene indicate statistically significant differences between groups (*p < *0.05). Values sharing at least one superscript letter are not significantly different.

Gene	Protein name	Fold change ± SEM (FBS10 = 1)			
		PS10	PS5+EGF+ITS	PS5+L‐WRN	FBS10
S100A4	S100 calcium‐binding protein A4 (S100A4/FSP1)	0.89 ± 0.05^b^	1.49 ± 0.08^c^	0.57 ± 0.11^a^	1.00 ± 0.04^bc^
SLC15A1	Peptide transporter 1 (PEPT1)	0.06 ± 0.03^a^	0.67 ± 0.12^a^	0.28 ± 0.25^a^	1.05 ± 0.23^a^
SLC5A1	Sodium/glucose cotransporter 1 (SGLT1)	0.63 ± 0.08^ab^	0.45 ± 0.05^a^	0.70 ± 0.03^ab^	1.02 ± 0.17^b^
SLC2A2	Glucose transporter 2 (GLUT2)	1.05 ± 0.30^b^	0.29 ± 0.08^a^	0.62 ± 0.05^ab^	0.82 ± 0.23^ab^
SLC27A4	Fatty acid transport protein 4 (FATP4)	0.68 ± 0.07^ab^	0.40 ± 0.04^a^	0.76 ± 0.04^b^	1.17 ± 0.28^b^
CD36	Cluster of differentiation 36 (CD36/FAT)	11.47 ± 3.71^b^	1.31 ± 0.10^a^	8.56 ± 0.15^b^	1.01 ± 0.12^a^
LAT2	L‐type amino acid transporter 2 (LAT2)	42.35 ± 7.21^bc^	185.96 ± 60.04^c^	6.07 ± 2.85^ab^	2.32 ± 1.95^a^
LAT4	L‐type amino acid transporter 4 (LAT4)	1.08 ± 0.24^ab^	0.33 ± 0.03^a^	1.56 ± 0.31^b^	1.02 ± 0.16^ab^
rBAT	Related to b0,+ amino acid transporter (rBAT)	0.76 ± 0.10^b^	0.26 ± 0.01^a^	1.03 ± 0.07^b^	0.96 ± 0.10^b^
ASCT2	Alanine–serine–cysteine transporter 2 (ASCT2)	0.65 ± 0.08^a^	0.65 ± 0.11^a^	0.94 ± 0.05^a^	1.00 ± 0.02^a^
SLC7A7	y^+^L amino acid transporter 1 (y^+^LAT1)	0.59 ± 0.07^b^	0.26 ± 0.05^a^	0.74 ± 0.03^b^	1.01 ± 0.13^b^
OCLN	Occludin (OCLN)	0.67 ± 0.04^a^	0.48 ± 0.03^a^	0.70 ± 0.08^a^	1.00 ± 0.05^b^
TJP1	Zonula occludens 1 (ZO1)	0.71 ± 0.01^b^	0.52 ± 0.02^a^	0.77 ± 0.06^bc^	1.01 ± 0.11^c^
CLDN2	Claudin‐2 (CLDN2)	77.53 ± 69.54^b^	1.61 ± 0.90^ab^	23.25 ± 1.21^b^	1.15 ± 0.37^a^
CLDN1	Claudin‐1 (CLDN1)	0.76 ± 0.07^ab^	0.49 ± 0.09^a^	0.79 ± 0.08^ab^	1.01 ± 0.12^b^
CLDN4	Claudin‐4 (CLDN4)	0.84 ± 0.17^a^	0.63 ± 0.08^a^	0.71 ± 0.04^a^	1.04 ± 0.27^a^
IL6	Interleukin‐6 (IL‐6)	0.66 ± 0.09^a^	0.64 ± 0.14^a^	0.74 ± 0.11^a^	1.00 ± 0.04^a^
IL8	Interleukin‐8 (IL8)	2.16 ± 0.47^a^	0.98 ± 0.29^a^	1.21 ± 0.21^a^	1.00 ± 0.03^a^
TNF	Tumor necrosis factor (TNF)	1.11 ± 0.46^a^	1.24 ± 0.44^a^	0.43 ± 0.08^a^	1.02 ± 0.15^a^
MUC1	Mucin‐1 (MUC1)	1.21 ± 0.02^b^	0.56 ± 0.04^a^	1.07 ± 0.05^b^	1.00 ± 0.08^b^
MUC2	Mucin‐2 (MUC2)	0.48 ± 0.07^a^	4.72 ± 2.56^ab^	7.11 ± 1.17^b^	1.03 ± 0.18^ab^

Among nutrient transporters, fatty acid transport protein 4 (SLC27A4, FATP4 [41]) and sodium‐glucose co‐transporter 1 (SLC5A1, SGLT1 [42]) mRNA transcript levels were lower in monolayers cultured in PS5+EGF+ITS (0.40 ± 0.04‐fold and 0.45 ± 0.05‐fold, respectively) than levels observed in monolayers in FBS10 (*p* > 0.05). The glucose transporter 2 (SLC2A2, GLUT2 [42]) mRNA was higher in monolayers in PS10 (1.05 ± 0.03‐fold vs. FBS10) compared with those cultured in PS5+EGF+ITS (0.29 ± 0.08‐fold vs. FBS10). For lipid uptake, cluster of differentiation 36 (CD36, FAT [41]) mRNA levels were significantly increased (*p < *0.05) in monolayers cultured in PS10 (11.47 ± 3.71‐fold) and PS5+L‐WRN (8.56 ± 0.15‐fold), in contrast to those cultured in FBS10 and PS5+EGF+ITS. LAT2 (neutral AA transporter at the basolateral membrane [43]) mRNA was higher (*p < *0.05) in monolayers in PS5+EGF+ITS (185.96 ± 60.04‐fold) and PS10 (42.35 ± 7.10‐fold), compared to monolayers in FBS10 media. Messenger RNA transcripts levels of SLC3A1 (rBAT, cationic and neutral AAs transporter at the apical membrane [43]) and SLC7A7 (y+LAT1, cationic AAs transporter at the basolateral membrane [43]) were lower (*p < *0.05) in monolayers in PS5+EGF+ITS (0.26 ± 0.01‐fold and 2.55 ± 0.05‐fold, respectively) compared to all other conditions. In addition, SLC43A2 (LAT4, neutral branched‐chain and aromatic AAs at the basolateral membrane [43]) mRNA was higher in monolayers with PS5+L‐WRN (1.56 ± 0.31‐fold vs. FBS10) compared to PS5+EGF+ITS (0.33 ± 0.03‐fold vs. FBS10; *p < *0.05).

Regarding epithelial barrier‐related biomarkers, *OCLN* mRNA levels were significantly lower in monolayers cultured in PS10 (0.67 ± 0.04‐fold), PS5+EGF+ITS (0.48 ± 0.03‐fold), and PS5+L‐WRN (0.70 ± 0.08‐fold) compared to those in FBS10 media (1.00 ± 0.05, *p < *0.05). Tight junction TJP1 (ZO1) and CLDN1 mRNA levels were significantly lower in monolayers cultured in PS5+EGF+ITS (0.52 ± 0.01‐fold and 0.59 ± 0.09‐fold, respectively; *p < *0.05) relative to those monolayers in FBS10. CLDN2 mRNA was significantly higher (*p* > 0.05) in IPEC–J2 cultured in PS5+L‐WRN (23.25 ± 1.21‐fold, *p < *0.05) and PS10 (77.53 ± 69.5‐fold, *p < *0.05) compared to FBS10 and PS5+EGF+ITS (1.61 ± 0.90‐fold).

For mucin expression, MUC2 mRNA was significantly higher in monolayers in PS5+L‐WRN (7.11 ± 1.17‐fold, *p < *0.05) compared to PS10 (0.48 ± 0.07). MUC1 mRNA was significantly lower in IPEC‐J2 cultured in PS5+EGF+ITS (0.56 ± 0.04‐fold) compared to P10, PS5+L‐WRN and FBS10 (*p < *0.05).

The biomarkers peptide transporter 1 (SLC15A1, PEPT1 [44]), alanine‐serine‐cysteine transporter 2 (SLC1A5, ASCT2 [43]), CLDN4, interleukin‐6 (*IL6*), interleukin‐8 (*IL8*), and tumor necrosis factor (*TNF*) [45] were similar in monolayers regardless of test media. The lineage‐specific markers CHGA (enteroendocrine cells [46]), CD163 (macrophages [47]), PTPRC (CD45, leukocytes [48]), as well as the AA transporters SLC6A19 (B^0^AT1), SLC6A20 (IMINO), SLC6A14 (ATB^0+^) were not detected in IPEC–J2 regardless of media conditions. In contrast, these markers were readily expressed in porcine jejunal tissue, with ΔCt values of CHGA = 5.93 ± 0.17, CD163 = 11.73 ± 1.07, CD45 = 4.17 ± 0.26, B^0^AT1 = 2.06 ± 0.24, IMINO = 3.98 ± 0.29, and ATB^0+^ = 12.47 ± 0.9. The fibroblast‐associated marker S100A4 [49] was expressed in all media conditions, with highest absolute levels in PS5+EGF+ITS (1.49 ± 0.08‐fold vs. FBS10). This marker was also present in tissue, but at significantly lower levels (Table S3; 0.03 ± 0.01‐fold vs. FBS10; *p* < 0.05).

Compared to porcine jejunal scrapings (Table S3), IPEC–J2 cultures showed markedly reduced expression of most absorptive and structural genes. PEP1 (220,011‐fold), SGLT1 (14,628‐fold), GLUT2 (3‐fold), FATP4 (307‐fold), CD36 (14‐fold), y+LAT1 (13‐fold), LAT2 (153,074‐fold), LAT4 (1150‐fold), and rBAT (45‐fold) mRNA were all substantially higher in tissue than in IPEC–J2 (FBS10; *p < *0.05). Messenger RNA levels of structural genes, such as CLDN2 and CLDN4, were elevated 389,089‐fold and 4.68‐fold, respectively, in tissue compared to IPEC–J2 cells (FBS10, *p* < 0.05). In contrast, ZO1 and CLDN1 mRNA were 0.41‐fold and 0.01‐fold lower in tissue, respectively, compared to FBS10 (*p* < 0.05). Interestingly, MUC2, IL8, and TNF mRNA were 1,212‐fold, 4.44‐fold, and 77.55‐fold higher in tissue, while MUC1 and IL6 mRNA were 0.02‐fold and 0.16‐fold lower, respectively (*p* < 0.05). The ASCT2 mRNA was not significantly different between tissue and FBS10 (*p* > 0.05).

### 3.5 Media Influences ZO1 Distribution and Cell Density in IPEC–J2 Monolayers

PS10 and PS5+EGF+ITS were selected for subsequent analyses, as they (1) ensued monolayers with the lowest yet stable TEER values at day 14; (2) supported enterocyte differentiation and sustained metabolic activity (ie ALP and LDH) (3) resulted in low OCLN and ZO1 expression levels consistent with a young gut [50] and (4) produced robust monolayers (ie resilient to HBSS buffer). Because HBSS is the buffer of choice for food bioavailability studies in polarized monolayers, maintenance of barrier integrity during incubation is an important attribute. Selecting both PS10 and PS5+EGF+ITS therefore, allowed inclusion of monolayers representing a broad and physiologically relevant range of paracellular permeability.

**FIGURE 4:**
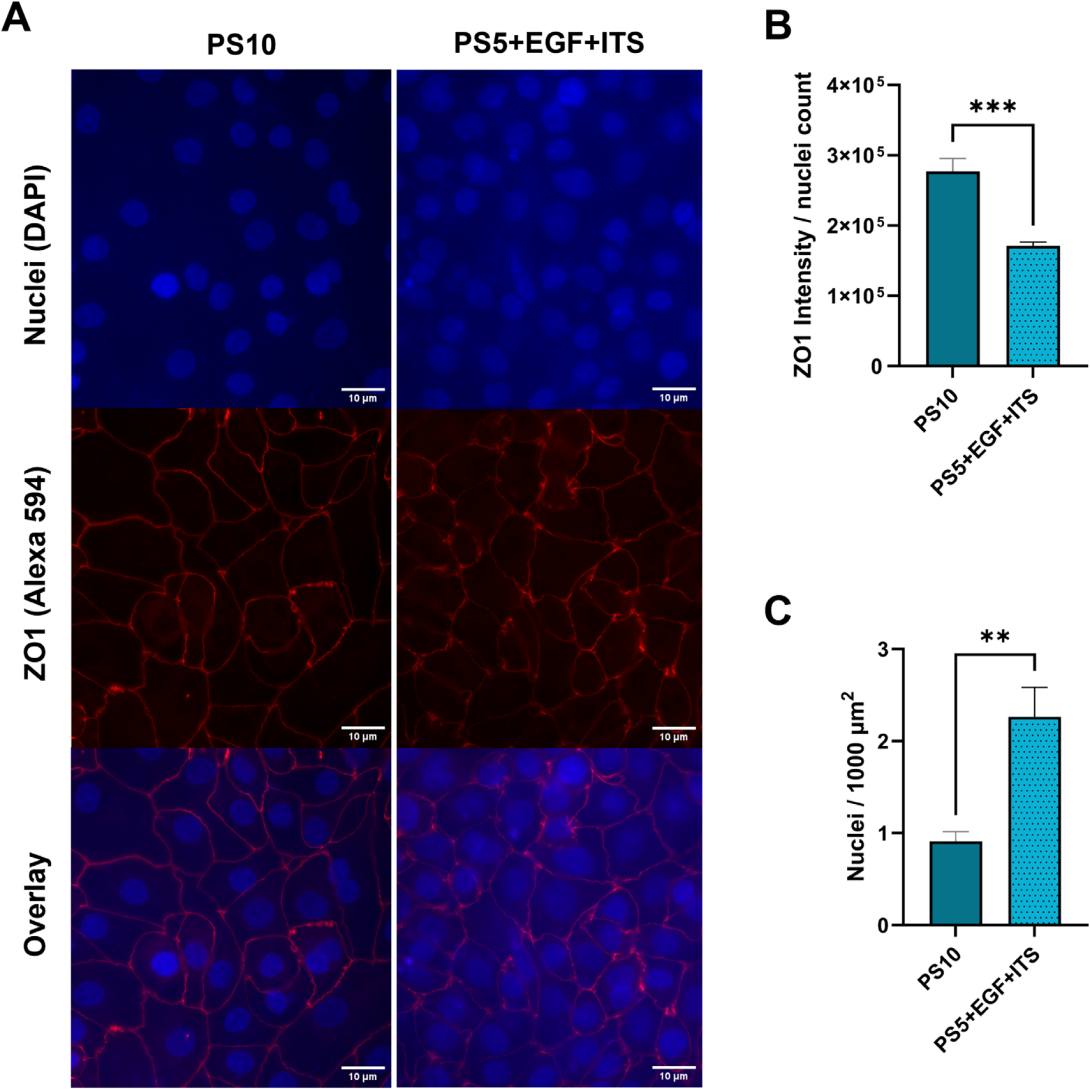
Effect of media composition on ZO1 localization and epithelial cell density in IPEC‐J2 monolayers. IPEC–J2 were cultured for 14 days in either PS10 or PS5+EGF+ITS medium. (A) Representative fluorescence microscopy images of monolayers stained for ZO1 (Alexa Fluor 555, red) and nuclei (DAPI, blue). Scale bar = 10 µm. (B) Fluorescence intensity of ZO 1 from images using the Otsu thresholding algorithm in Fiji‐ImageJ and normalized to the number of nuclei. (C) Nuclei count per 1000 µm^2^ is used as a proxy for cell density. Results represent the average of three biological replicates with three non‐overlapping images per replicate (mean ± SEM). Statistical differences were evaluated using unpaired t‐tests (*p < *0.05).

To investigate the impact of PS10 and PS5 +EGF/ITS on tight junction architecture, ZO1 immunofluorescence staining was performed on day 14 monolayers (Figure 4). ZO1 is a tight junction protein that links claudins and occludins to the actin cytoskeleton and regulates epithelial barrier integrity and paracellular permeability [51]. Representative images of ZO1 staining in monolayers grown in PS10 and PS5+EGF+ITS conditions are depicted in Figure 4A. Quantification of the raw integrated density of the ZO1 signal, normalized to the number of nuclei, revealed significantly higher expression in monolayers cultured in PS10 compared to PS5+EGF+ITS (277,086 ± 18,412 vs. 171,298 ± 5,215raw IntDen/ nuclei count, respectively; *p < *0.05; Figure 4B). Interestingly, the cell density, expressed as nuclei per 1000 µm^2^, was significantly greater in monolayers with PS5+EGF+ITS compared to PS10 (2.26 ± 0.32 vs. 0.91 ± 0.11; *p < *0.05; Figure 4C), suggesting a higher cell proliferation or seeding efficiency in this condition. Taken together, there were more cells in the monolayers cultured in PS5+EGF+ITS, but more ZO1 in monolayers cultured in PS10.

### 3.6 IPEC–J2 Viability Is Maintained After Exposure to Inactivated Digesta

To assess the biocompatibility of IPEC‐J2 monolayers with digested food, infant formula was first subjected to static gastrointestinal digestion using the INFOGEST protocol mimicking the infant gut. At the end of the intestinal phase, digesta was detoxified by thermal inactivation or inhibition of digestive enzymes. These inactivation strategies were selected as commonly used approaches to detoxify digested samples for cell culture applications [52]. Heat inactivation effectively halts digestive enzyme activity but may cause structural changes to digesta and may destroy bioactivity. Alternatively, chemical inhibition (P+O) will preserve structure and bioactivity of digesta but may interfere with cell monolayer function. As exposure of IPEC–J2 cells to digesta remains limited in the literature, both methods were evaluated for biocompatibility with IPEC–J2. Digesta samples were also diluted to dilute bile salts, with the digesta concentration applied to monolayers ranging from 400 to 100 µg protein/cm^2^. Successful digestion was evidenced by disappearance of high molecular weight bands on SDS‐PAGE and increases in free amino groups as measured by o‐phthaldialdehyde (OPA) assay (Figure S1). A significant increase in free amino groups was observed from G0 (pre‐gastric phase) to I60 (post‐intestinal phase) (*p < *0.05), confirming successful digestion.

**FIGURE 5:**
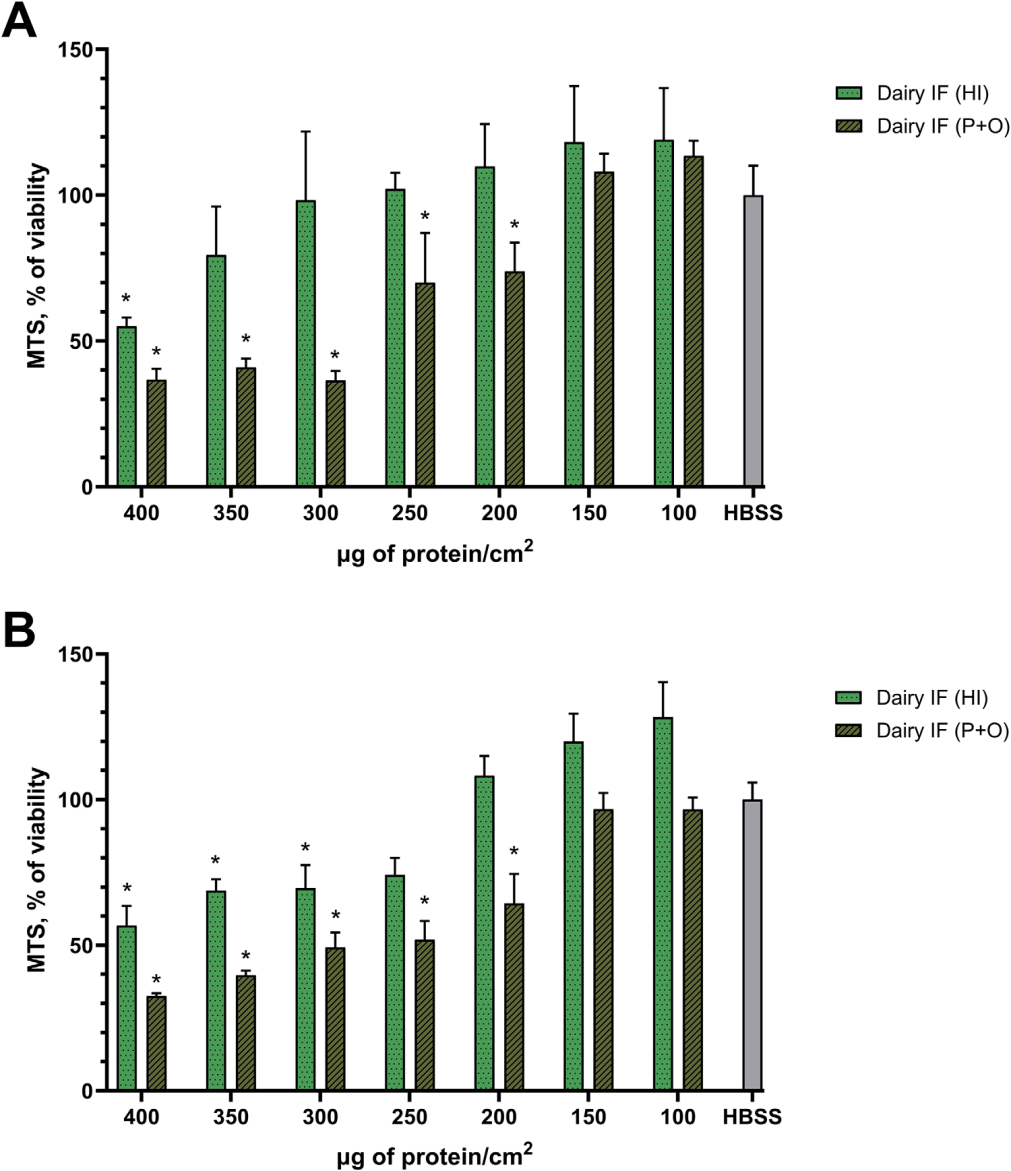
Viability of undifferentiated IPEC–J2 cells exposed to dairy infant formula (IF) digesta. Dairy IF was subjected to simulated infant gastrointestinal digestion and inactivated by either heat inactivation (HI) or by Pefabloc and Orlistat (P+O). Undifferentiated IPEC–J2 cells were cultured for 24 h in either PS10 or PS5+EGF+ITS medium. Cells were then treated with dairy IF digesta for 2 h and viability assessed using MTS. Results were normalized to cells in HBSS control (100% viable). (A) PS10‐cultured cells treated with a range of concentrations of dairy IF (HI) or (P+O) digesta. (B) PS5+EGF+ITS‐cultured cells treated with a range of concentrations of dairy IF (HI) or (P+O) digesta. Data are presented as mean ± SEM from three independent experiments, in technical duplicates. Statistical analysis was performed using two‐way ANOVA followed by Dunnett's multiple comparisons test to compare treated groups with HBSS control at each concentration. *p < *0.05 was considered statistically significant and is indicated by an asterisk. Cell viability in the HBSS control corresponded to 84.26 ± 6.10% of that observed in cells maintained in PS10 or PS5+EGF+ITS medium alone.

To study the tolerance of IPEC‐J2 cells to digested dairy IF, digesta at various protein concentrations were applied to undifferentiated IPEC‐J2 cells cultured overnight in PS10 or PS5+EGF+ITS, and cell viability assessed by MTS assay (Figure 5). For cells cultured in PS10 medium, a 2 h treatment with dairy IF (HI), at <350 µg protein /cm^2^, showed no significant difference in viability compared to the cells in HBSS buffer (Figure 5A; *p* > 0.05). Inactivation of digestive enzymes via P+O, was less biocompatible with IPEC‐J2 cells. In this instance, dairy IF P+0 at <150 µg protein/cm^2^ was safe for use on IPEC‐J2 cells. For PS5+EGF+ITS medium, IPEC‐J2 cells treated for 2 h with 250 µg/cm^2^ for dairy IF (HI) or150 µg/cm^2^ for dairy IF (P+O) had similar viability to cells in HBSS for 2 h (96.66 ± 5.58%; *p* > 0.05). Digesta control samples, where H_2_O is used in the digestion rather than dairy IF, were non‐toxic to IPEC‐J2 cells at any dilution tested (*p* > 0.05 compared to cells in HBSS).

### 3.7 Heat‐Inactivated Digested Infant Formula—Dairy IF (HI)—Increase TEER in IPEC–J2 Monolayers Cultured in PS10 Medium

**FIGURE 6:**
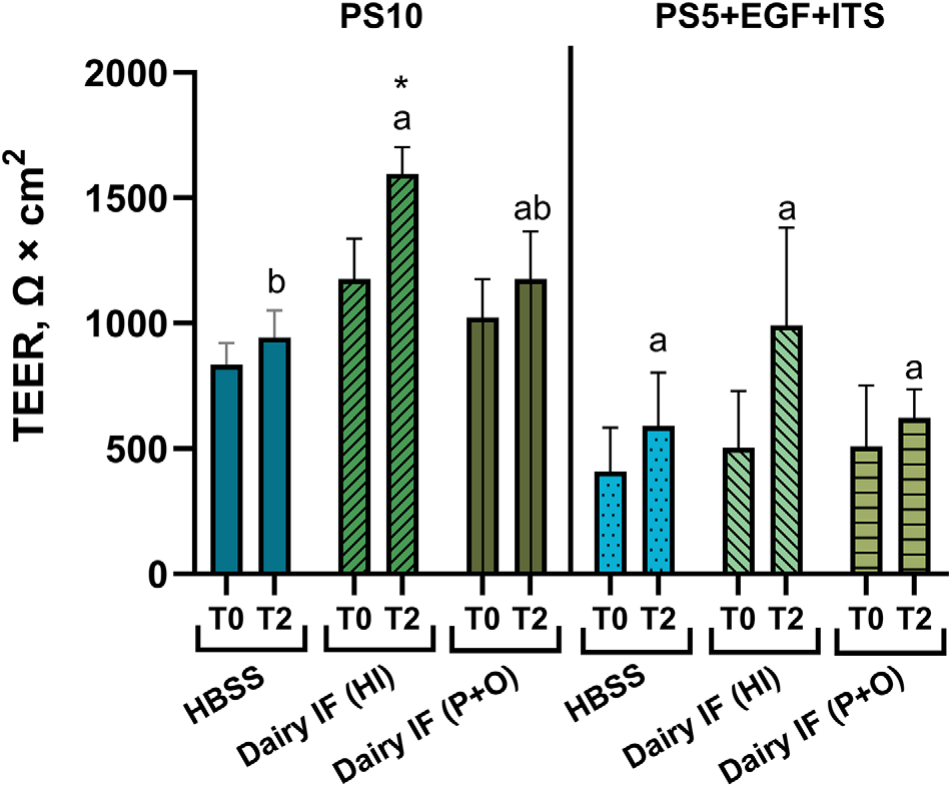
TEER values of undifferentiated IPEC–J2 cells before and after exposure to dairy infant formula (IF) digesta. IPEC–J2 cells were cultured for 14 days in either PS10 or PS5+EGF+ITS medium and then treated for 2 h with digested dairy IF detoxified with heat treatment (HI) or with Pefablock and Orlistat (P+O). Transepithelial electrical resistance (TEER) was measured at T0 (after washing monolayers, immediately before digesta addition) and T2 (2 h post‐treatment). Dairy IF (H1) was added at 250 µg protein/cm^2^. Dairy IF (P+O) was added at 150 µg protein/cm^2^. Data are presented as mean ± SEM from three independent experiments and technical duplicates. Statistical analysis was performed separately for each medium using one‐way ANOVA with Tukey's post hoc test; different letters indicate significant differences between treatments within the same time point (*p < *0.05). Student's t‐tests were used to compare T0 and T2 within each condition, with asterisks indicating significant differences.

To assess barrier integrity after treatment with digested dairy IF, IPEC–J2 monolayers were cultured for 14 days in PS5+EGF+ITS or PS10 medium and then treated with digested dairy IF for 2 h. Monolayer TEER was measured at baseline (T0) and after 2 h (T2, Figure 6). Monolayers cultured in PS10 exhibited a significant increase in TEER after treatment with dairy IF (HI) (T0 = 1175.5 ± 162.7 vs. T2 = 1595.7 ± 107.0 Ω × cm^2^; *p < *0.05). At T2, these monolayers also exhibited significantly higher TEER compared with HBSS controls (HBSS = 942.15 ± 109.25 Ω × cm^2^; *p < *0.05). When these monolayers were treated with dairy IF (P+O), TEER remained unchanged from T0 to T2 (T0 = 1023 ± 153.66 vs. T2 = 1175.90 ± 189.68 Ω* × *cm^2^; *p* > 0.05). Where monolayers were cultured in PS5+EGF/IT medium for 14 days, TEER values remained stable pre‐ and post‐treatment with dairy IF (HI) and dairy IF P+0 (*p* > 0.05).

### 3.8 PS10 Increase Basolateral Free AA After Exposure to Dairy IF Digesta

**FIGURE 7:**
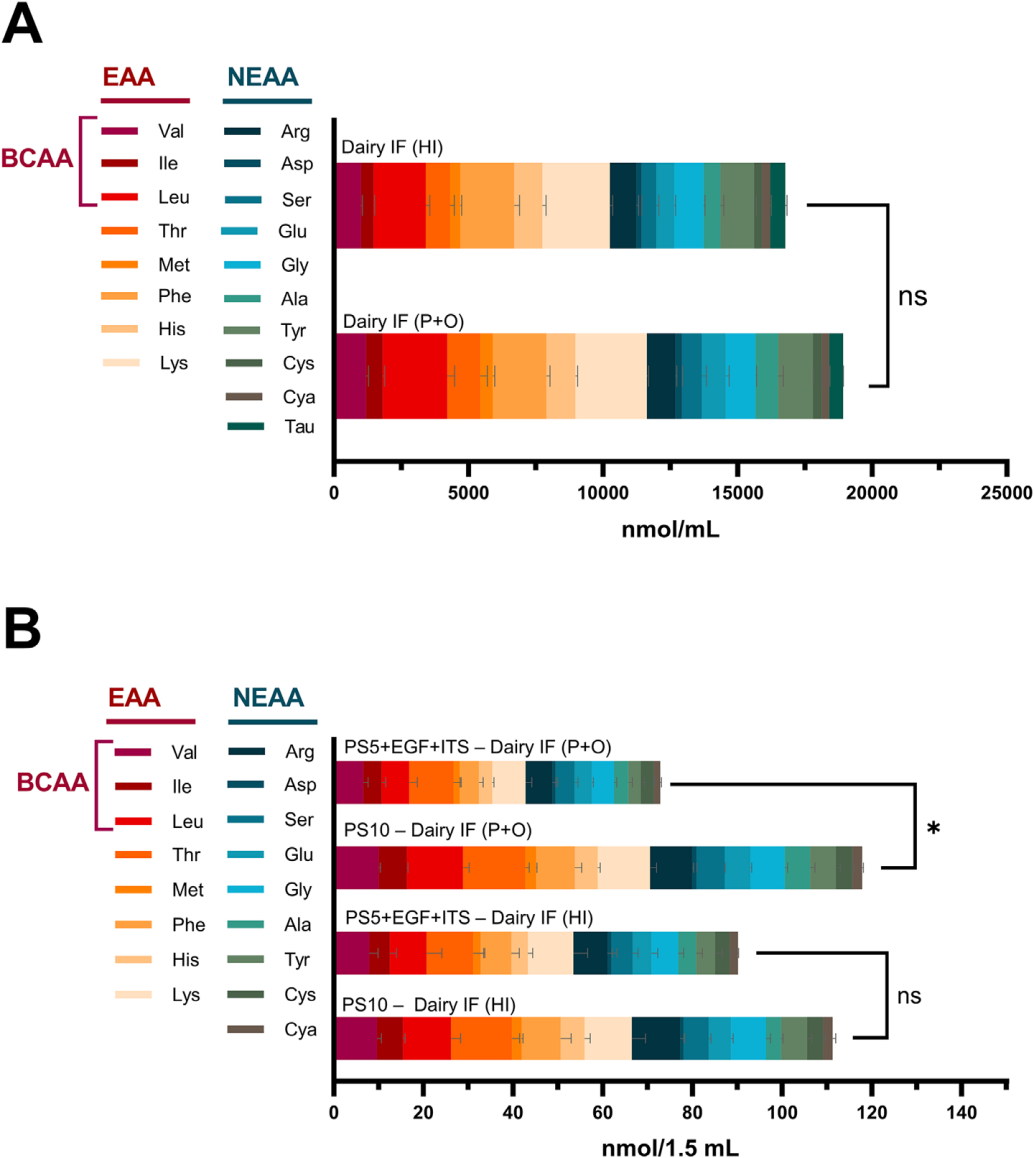
Free amino acid (AA) in basolateral chamber of IPEC–J2 monolayers treated with dairy infant formula (IF) digesta. IPEC–J2 monolayers were cultured for 14 days in either PS10 or PS5+EGF+ITS medium and then exposed for 2 h to dairy IF digesta (dairy IF) inactivated by heat (HI; 200 µg protein/cm^2^ in HBSS) or by Pefabloc and Orlistat (P+O; 150 µg protein/cm^2^ in HBSS). (A) Total free AA content in the undiluted digesta. (B) Free AAs in the basolateral compartment. Colors indicate AA classification: red = branched‐chain AAs (BCAA), orange/red = essential AAs (EAA), and blue/green = nonessential AAs (NEAA). Cya = cysteic acid, oxidation product of cysteine. Data represent mean ± SEM from three independent experiments and technical duplicates. Statistical analysis was performed using unpaired t‐tests. Asterik indicates significant difference (*p < *0.05) between PS5+EGF+ITS and PS10 within each digesta condition, whereas ‘ns’ denotes not significant.

Next, the concentration of free AAs, in the basolateral chambers of monolayers, was quantified to assess media influence on the absorption capability of IPEC‐J2 monolayers to digested dairy IF. Figure 7A shows the free AA concentration in the dairy IF (HI) (1838 ± 34.85 nmol/mL) was not significantly different to dairy IF (P+O) (2065 ± 96.81 nmol/mL; *p* > 0.05). Significant differences were observed in the basolateral compartment of IPEC‐J2 monolayers cultured with either PS10 or PS5+EGF+ITS, and subsequently treated with either dairy IF (HI) or dairy IF (P+O) (Figure 7B). Monolayers cultured in PS10 showed higher total free AA concentrations after treatment with dairy IF (P+O) compared to monolayers in PS5+EGF+ITS (117.8 ± 7.90 vs. 72.82 ± 12.53 nmol/1.5 mL; *p < *0.05). This was also evident for essential AAs (PS10 = 70.53 ± 4.81 vs. PS5+EGF+ITS = 49.79 ± 8.19 nmol/1.5 mL; *p < *0.05) and branched‐chain AAs (PS10 = 28.79 ± 1.61 vs. PS5+EGF+ITS = 16.81 ± 3.84 nmol/1.5 mL; *p < *0.05). No significant differences were observed in free AAs levels in IPEC‐J2 basolateral chambers, between media types following dairy IF (HI) treatment (PS10 = 111.3 ± 15.1; PS5 +EGF/ITS = 90.17 ± 25.05 nmol/1.5 mL; *p < *0.05). Tables S4 and S5 report the concentration of each single AA in the digested samples and basolateral compartment, respectively.

Following 2 h of incubation, the apical compartment of IPEC‐J2 retained similar levels of free AAs across all conditions (Figure S2): PS10 dairy IF (HI) = 757.5 ± 64.34, PS5+EGF+ITS dairy IF (HI) = 738.6 ± 23.22, PS10 dairy IF (P+O) = 511.3 ± 40.76, PS5+EGF+ITS dairy IF (P+O) = 499.1 ± 50.02 nmol/0.5 mL (*p < *0.05).

Although the dairy IF‐derived AA were not labelled, Figure S2 presents (B) apical‐to‐basolateral ratios illustrating AA distribution across the monolayer and (C–D) apical and basolateral concentrations in HBSS‐treated controls. Baseline AA levels in HBSS‐treated monolayers were low and comparable between conditions (apical: PS10 = 12.57 ± 1.33 and PS5+EGF+ITS = 13.60 ± 1.73 nmol/0.5 mL; basolateral: PS10 = 33.42 ± 6.93 and PS5+EGF+ITS = 33.06 ± 10.88 nmol/0.5 mL; ns). Treatment of day 14 IPEC‐J2 monolayers cultured in PS10 with 150 µg protein/cm^2^ dairy IF P+0 resulted in significantly enhanced basolateral‐to‐apical AA ratios, indicating greater transfer compared with PS5+EGF+ITS. In particular, branched‐chain AAs (Val, Ile, Leu) and Thr, as well as non‐essential AAs (Arg, Ser, Gly, Ala, and Cysteic acid), showed markedly higher basolateral/apical ratios in monolayers grown in PS10 (*p < *0.05).

## 4 Discussion

Previously, IPEC‐J2 monolayers were cultured under diverse conditions, such as FBS [22, 30, 53], PS [22], or growth factor–supplemented formulations, such as EGF/ITS [20, 22, 32, 33] or L‐WRN conditioned media [34]. This has contributed to wide differences in reported TEER values from monolayers and limited reproducibility between laboratories.

In the present study, four media were compared: (1) PS10, a species‐specific media known to produce monolayers with physiologically relevant TEER values (∼300–500 Ω* × *cm^2^ [22]); (2) PS5+EGF+ITS, reported to maintain low TEER (240–330 Ω* × *cm^2^) together with epithelial morphology resembling native jejunum [22]; (3) PS5+L‐WRN, where supplementation with Wnt3a, R‐spondin, and Noggin reduces TEER to ∼50 Ω* × *cm^2^ but promotes IPEC–J2 proliferation [34] and (4) FBS10 recommended by the supplier but known to yield monolayers with very high and variable TEER (2000–15 000 Ω cm^2^ [22, 53]. The aim was to identify one media that provides a reproducible balance of barrier integrity, differentiation, and nutrient absorption, thereby advancing IPEC–J2 as a standardized and physiologically relevant model for infant nutrition research.

In the assessment of the four media types, our primary findings are that (a) monolayer TEER values varied substantially with culture media, ranging from 327.58 ± 71.5 Ω* × *cm^2^ in PS5+EGF+ITS to 6620.78 ± 1997.03 Ω* × *cm^2^ in FBS10; however, this did not overly influence monolayer paracellular or transcellular permeability; (b) media composition strongly influenced the expression of IPEC–J2 nutrient transporters and tight junction proteins and was associated with distinct cellular traits, with PS5+EGF+ITS encouraging more mesenchymal‐like characteristics in IPEC–J2, while FBS10 and PS5+L‐WRN supported IPEC–J2 tight barriers but with reduced resilience.

PS10 emerged as the most balanced condition. Monolayers cultured in PS10 displayed TEER values = 2395.0 ± 485.5 Ω × cm^2^, high ALP and LDH activity (8.01 ± 1.54 IU/mg of protein and 0.12 ± 0.01 mU/mg of protein), and the lowest lactulose permeability (Papp = 9.66 × 10^−^^7^ ± 1.97 × 10*^−^* ^7^ cm/s). Immunofluorescence indicated that ZO1 displayed an organized distribution at the cell membrane, and mRNA levels of CD36, LAT2, and CLDN2 more closely resembled pig jejunum. Moreover, S100A4 expression was lower than in PS5+EGF+ITS and PS5+L‐WRN, consistent with a less fibroblast‐like phenotype. Functionally, monolayers cultured in PS10 were compatible with both HBSS buffer and digested dairy IF, showing a stable TEER or even a TEER increase after a 2 h treatment with dairy IF (HI). These monolayers had a substantial concentration of free AAs in their basolateral chambers post dairy IF apical treatment, significantly higher than monolayers cultured in PS5+EGF+ITS (*p < *0.05). These findings highlight PS10 as the most suitable culture condition for generating robust and functional IPEC‐J2 monolayers for food absorption studies.

In agreement with earlier reports, culturing IPEC‐J2 in FBS10 yielded the highest TEER values, comparable to those described by Zakrzewski et al. (2013), Saaby et al. (2016), and Bernardini et al. (2021). Zakrzewski et al. (2013) observed a monolayer TEER of 2111 ± 356 Ω × cm^2^ in 10% FBS and only 241 ± 28 Ω × cm^2^ in 5% PS+EGF/ITS [22]. Similarly, Saaby et al. recorded 15,833 ± 1596 Ω × cm^2^ for monolayers cultured in 10% FBS, along with a mannitol Papp of 0.04 × 10*^−^* ^6^ cm/s [53]. Bernardini et al. reported lower TEER values of approximately ∼4500 Ω × cm^2^ for monolayers in 10% FBS and ∼1550 Ω × cm^2^ for 5% FBS [30].

In our study, remarkably, IPEC‐J2 cultured in PS5+L‐WRN performed similarly to culturing in FBS10, despite lacking bovine serum. This likely reflects the effects provided by Wnt3a, R‐spondin, and Noggin, which are known to promote cell proliferation by activating canonical Wnt signaling pathways [54]. In contrast, PS10 alone supported monolayers with moderate TEER (2395.00 ± 485.51 Ω × cm^2^), whereas PS5+EGF+ITS media produced monolayers with the weakest barrier impedance (327.58 ± 71.58 Ω × cm^2^). Notably, real‐time CellZScope analysis confirmed that monolayer TEER plateaued around day 12 for all conditions, suggesting that epithelial barrier maturation was complete by this time point.

Moreover, in the present study, IPEC‐J2 monolayers exhibited a marked TEER decrease following exposure to lactulose:mannitol in HBSS. Monolayers cultured in FBS10 and PS5+L‐WRN showed the greatest TEER loss, whereas those cultured in PS5+EGF+ITS were the least affected. In contrast, Perruchot et al. (2025) reported a significant TEER reduction in IPEC–J2 monolayers cultured in PS5+EGF+ITS after HBSS washing (from 3100 ± 684 Ω × cm^2^ to 1342 ± 297 Ω × cm^2^; *p < *0.05) [35]. Importantly, a decline in TEER did not necessarily indicate increased permeability: lactulose and mannitol fluxes were highest in PS5+EGF+ITS, followed by PS5+L‐WRN and FBS10, confirming that higher TEER values do not always correspond to reduced paracellular transport. This divergence may be partially explained by differences in epithelial differentiation and metabolic activity. ALP activity, a marker of enterocyte maturation [39], and LDH activity, indicative of glycolytic metabolism [55], were both higher in IPEC‐J2 cultured in PS10 and PS5+EGF+ITS compared to FBS10 and PS5+L‐WRN (*p < *0.05). Together, these findings suggest that although FBS10 and PS5+L‐WRN support the formation of IPEC–J2 tight epithelial barriers, as reflected by high baseline TEER, they may preferentially encourage cell proliferation over maturation. In contrast, PS10 and PS5+EGF+ITS promoted lower TEER values on day 14 IPEC–J2 but higher ALP and LDH activity, indicating greater metabolic activity, enhanced differentiation, and potentially a more physiologically relevant epithelial phenotype.

Gene expression and structural characterization of IPEC‐J2 under different media revealed distinct phenotypes ranging from proliferative to differentiated epithelial states. In PS10, GLUT2 and CD36 mRNA were higher than in PS5+EGF+ITS, while CD36 and LAT2 were elevated compared with FBS10; CLDN2 was also increased relative to both FBS10 and PS5+EGF+ITS (*p < *0.05), indicating enhanced nutrient transport capacity. In PS5+L‐WRN, CD36 was higher than in FBS10 or PS5+EGF+ITS, and GLUT2 and LAT4 were increased relative to PS5+EGF+ITS; CLDN2 was higher than in FBS10, and MUC2 exceeded levels in PS10 (*p < *0.05), reflecting additional goblet‐like features. In contrast, PS5+EGF+ITS monolayers showed the highest S100A4 but reduced FATP, SGLT1, rBAT, y^+^LAT1, and MUC1 compared with all other media, while LAT2 was higher than FBS10 (*p < *0.05). This mRNA pattern is consistent with a fibroblast‐like, less differentiated phenotype. Finally, FBS10 cultures expressed higher OCLN, ZO1, and CLDN1 than any other medium (*p < *0.05), whereas CD36 and LAT2 were lower than in PS10 and PS5+EGF+ITS, respectively, indicating greater barrier integrity but reduced nutrient transporter activity.

To validate these transcriptional trends at the protein level, ZO1 immunofluorescence staining revealed significantly greater membrane‐associated ZO1 signal in monolayers cultured in PS10 compared to PS5+EGF+ITS. Conversely, nuclear density was significantly greater in PS5+EGF+ITS than in PS10 (*p < *0.05), consistent with increased proliferative activity in the PS5+EGF+ITS‐based condition.

The only published study directly comparing different media in IPEC‐J2 is Zakrzewski et al. (2013), who reported that CLDN4 was significantly upregulated (*p < *0.05) in IPEC‐J2 under PS5+EGF+ITS compared to FBS10, while CLDN2 was absent at both the mRNA and protein level. For us, CLDN4 was detectable under all tested conditions but not significantly different (*p* > 0.05), while CLDN2 expression was clearly present and significantly increased only in monolayers cultured in PS10‐based media (*p < *0.05) compared to FBS10. Zakrzewski et al. also reported significantly higher protein levels of GLUT2 (*p < *0.01), SGLT1 (*p < *0.05), and the mesenchymal marker vimentin (*p < *0.01) in cells grown in PS5+EGF+ITS compared to FBS10‐grown cells. In our study, SGLT1 and GLUT2 mRNA were not significantly different between conditions, with SGLT1 expressed at a low level (approximately 20,000‐fold lower than in jejunal tissue). While Zakrzewski identified vimentin as a mesenchymal marker upregulated in IPEC–J2 with PS5+EGF+ITS, we observed S100A4 to be similarly expressed in monolayers in either FBS10 or PS5+EGF+ITS media. Nevertheless, expression remained 13‐ to 37‐fold higher than in jejunal tissue across all conditions (*p < *0.05), indicating a persistent mesenchymal‐like phenotype in vitro. Regarding mucins, their study showed MUC2 was undetectable in monolayers cultured in FBS but inducible with PS, which is consistent with our observation that MUC2 mRNA was significantly upregulated in monolayers with PS5+EGF+ITS compared to FBS10.

When compared to porcine jejunum, all in vitro conditions showed markedly reduced mRNA transcripts (*p < *0.05) of nutrient transporters (PEP1, SGLT1, GLUT2, FATP4, LATs, rBAT, CD36) structural genes (CLDN2, CLDN4), mucin and cytokine genes (MUC2, IL8, TNF), while ZO1, CLDN1, MUC1, and IL6 were higher (*p < *0.05) in culture. Furthermore, immune, lineage‐specific markers and AA transporters such as CHGA, CD163, CD45, B0AT1, IMINO, and ATB0+ were absent from IPEC–J2 cultures but readily detected in tissue, underscoring the restricted cell‐type diversity of this model. Thus, although media composition strongly influences the balance between barrier, absorptive, and secretory phenotypes in IPEC–J2, none of the in vitro conditions fully replicate the transcriptional complexity of jejunal epithelium.

These observations are further contextualized by the findings of Støy et al. (2013), who compared gene expression profiles between IPEC–J2 cells and preterm pig jejunal tissue under colostrum or formula feeding [56]. Their principal component analysis (PCA) revealed a clear separation between IPEC‐J2 and native tissue, indicating substantial transcriptional divergence. Notably, Støy et al. (2013) reported that MUC2 and ALPI were not expressed in IPEC‐J2 under any tested condition. In contrast, we detected low but consistent expression of MUC2. Importantly, while ALPI transcript levels were not assessed here, we identified clear ALP enzymatic activity across all culture conditions, with higher activity in PS10‐ and PS5+EGF+ITS‐cultured monolayers. These discrepancies likely reflect known variability in IPEC–J2 phenotype, including phenotypic drift and genomic instability (e.g., aneuploidies and structural rearrangements) [57], as well as differences in culture conditions, passage history, and cell handling between studies.

Regarding immune‐related genes, both our data and Støy et al. confirmed expression of IL8, IL6, and TNF in IPEC‐J2, with IL6 consistently higher in IPEC–J2 than in jejunal tissue [56].

A recent study characterized the genomic architecture of IPEC‐J2 and reported multiple chromosomal abnormalities, including whole‐chromosome and segmental aneuploidies [57]. Notably, chromosome 9 was found to be fully triploid, which encompasses both IL6 (SSC9: ∼91.5 Mb) and CD36 (SSC9: ∼99.7 Mb). In addition, the distal half of chromosome 8, which includes IL8 (SSC8: ∼69.9 Mb), showed a clear copy number gain. While the IL6 pattern is consistent with the dosage effects of chromosomal gains, these results also indicate that additional mechanisms, such as altered chromatin accessibility and epigenetic regulation, further influence transcriptional outcomes in this model [57].

Similar discrepancies have also been reported in other well‐established intestinal cell lines. For example, Sun et al. (2002) demonstrated that Caco‐2 cells diverge markedly from human duodenal tissue, with SGLT1 expressed more than 600‐fold higher in the duodenum, while GLUT3 was 150‐fold higher in Caco‐2 [58]. In addition, differences in developmental stage may also contribute to the observed discrepancies, as the jejunal samples were obtained from post‐weaning piglets rather than neonates.

The suitability of IPEC–J2 monolayers to food digesta studies was demonstrated with dairy IF. From MTS and TEER data, it was evident that heat inactivation with dilution of digested dairy IF was preferable to the use of protease inhibitors. Indeed, previous studies have reported adverse effects of Pefabloc on Caco‐2 epithelial integrity [52], supporting the interpretation that enzymatic inhibitors are harmful. Consistent with our findings, Perruchot et al. (2025) also reported no significant TEER change in IPEC–J2 monolayers after 2 h exposure to heat‐inactivated digested pea flour (*p* > 0.05) [35].

Quantification of free AAs in the basolateral chambers of IPEC‐J2 monolayers treated with digested dairy IF showed that dairy IF (P+O) treatment enhanced basolateral AA transport in PS10‐grown cells compared with PS5+EGF+ITS, consistent with higher expression of apical (rBAT) and basolateral (y^+^LAT1) transporters and a more functionally differentiated epithelial phenotype. To our knowledge, this is among the pioneering studies exposing IPEC‐J2 monolayers to digested food matrices, demonstrating that PS10‐cultured monolayers maintain barrier integrity and nutrient transport following exposure to infant formula digesta.

Despite the valuable insights gained from this study, several limitations warrant consideration. Passage number evaluation was not possible in our study because this information is not provided by the cell culture collection. Comparison with jejunal tissue should be interpreted with caution, as samples were obtained from post‐weaning piglets rather than neonates. Although transcript‐level analyses were performed, protein‐level validation was limited to ZO1, and additional protein‐based analyses would further strengthen the mechanistic interpretation of the findings. In addition, free AA analysis provides only a snapshot of AA distribution between compartments and does not distinguish between transepithelial transport, cellular metabolism, or intracellular utilization for protein synthesis. Future studies using labelled AAs would allow a more precise assessment of transport dynamics and cellular utilization. Last, our study focused on a relatively narrow set of nutrient types and digestion states; expanding the analysis to include additional nutrient classes—such as carbohydrates, vitamins, or essential minerals—would further enhance the translational relevance of the model.

## 5 Conclusions

In conclusion, culture medium composition markedly affected IPEC–J2 barrier integrity, differentiation, and responses to digested food. Among the tested conditions, PS10 provided the most physiologically relevant balance, supporting moderate yet stable TEER, elevated ALP and LDH activity, and tighter junctional organization with reduced S100A4 expression—indicating lower fibroblast‐like activity and greater epithelial differentiation. These characteristics suggest that PS10‐grown monolayers best replicate the functional balance between barrier selectivity and nutrient absorption observed in vivo. The expression of key nutrient transporters and tight junction proteins further validates this model for studying nutrient transport and gut barrier responses. Finally, heat inactivation proved preferable to enzyme inhibition for digesta biocompatibility. Together, these results establish IPEC–J2 as a robust, non‐cancerous alternative to Caco‐2 for evaluating dietary protein absorption and epithelial resilience, particularly in the context of infant nutrition.

## Author Contributions

**Francesca Bietto**: conceptualization, data curation, formal analysis, investigation, methodology, visualization, validation, writing – original draft, writing. **Myriam M.‐L. Grundy**: conceptualization, investigation, methodology, validation, writing – review and editing. **Elena Arranz**: methodology, supervision, validation, writing – review and editing. **Eva Rath**: methodology, supervision, validation, writing – review and editing. **Alice J. Lucey**: methodology, supervision, validation, resources, writing – review and editing. **Linda Giblin**: conceptualization, methodology, supervision, funding acquisition, validation, writing – review and editing, project administration, resources.

## Funding

This work was supported by a Teagasc Walsh Scholarship (2021007).

## Conflicts of Interest

The authors declare no conflicts of interest.

## Supporting information




**Supporting File**: mnfr70540‐sup‐0001‐SuppMat.docx.

## Data Availability

The data that support the findings of this study are available from the corresponding author upon reasonable request.

## References

[1] C. Chelakkot , J. Ghim , and S. H. Ryu , “Mechanisms Regulating Intestinal Barrier Integrity and its Pathological Implications,” Experimental & Molecular Medicine 50 (2018): 1–9. 10.1038/s12276-018-0126-x PMC6095905 30115904

[2] T. Otani and M. Furuse , “Tight Junction Structure and Function Revisited,” Trends in Cell Biology 30 (2020): 805–817. 32891490 10.1016/j.tcb.2020.08.004

[3] P. Paone and P. D. Cani , “Mucus Barrier, Mucins and Gut Microbiota: The Expected Slimy Partners?,” Gut 69 (2020): 2232–2243. 32917747 10.1136/gutjnl-2020-322260 PMC7677487

[4] P. R. Kiela and F. K. Ghishan , “Physiology of Intestinal Absorption and Secretion,” Best Practice & Research Clinical Gastroenterology 30 (2016): 145–159. 27086882 10.1016/j.bpg.2016.02.007 PMC4956471

[5] P. T. Sangild , D. M. Ney , D. L. Sigalet , A. Vegge , and D. Burrin , “Animal Models of Gastrointestinal and Liver Diseases. Animal Models of Infant Short Bowel Syndrome: Translational Relevance and Challenges,” American Journal of Physiology‐Gastrointestinal and Liver Physiology 307 (2014), G1147–G1168. 25342047 10.1152/ajpgi.00088.2014 PMC4269678

[6] T. Lea , The Impact of Food Bioactives on Health: In Vitro and Ex Vivo Models, ed. K. Verhoeckx , P. Cotter , I. López‐Expósito , et al. (Springer International Publishing, 2015), 103–111. 29787039

[7] H. Sun , E. C. Chow , S. Liu , Y. Du , and K. S. Pang , “The Caco‐2 Cell Monolayer: Usefulness and Limitations,” Expert Opinion on Drug Metabolism & Toxicology 4 (2008): 395–411. 18433344 10.1517/17425255.4.4.395

[8] B. Srinivasan , A. R. Kolli , M. B. Esch , H. E. Abaci , M. L. Shuler , and J. J. Hickman , “TEER Measurement Techniques for In Vitro Barrier Model Systems,” SLAS Technology 20 (2015): 107–126. 10.1177/2211068214561025 PMC4652793 25586998

[9] Y. Sambuy , I. Angelis , G. Ranaldi , M. L. Scarino , A. Stammati , and F. Zucco , “The Caco‐2 Cell Line as a Model of the Intestinal Barrier: Influence of Cell and Culture‐Related Factors on Caco‐2 Cell Functional Characteristics,” Cell Biology and Toxicology 21 (2005): 1–26. 15868485 10.1007/s10565-005-0085-6

[10] I. Hubatsch , E. G. E. Ragnarsson , and P. Artursson , “Determination of Drug Permeability and Prediction of Drug Absorption in Caco‐2 Monolayers,” Nature Protocols 2 (2007): 2111–2119. 17853866 10.1038/nprot.2007.303

[11] C. Catassi , A. Bonucci , G. V. Coppa , A. Carlucci , and P. L. Giorgi , “Intestinal Permeability. Changes During the First Month,” Journal of Pediatric Gastroenterology and Nutrition 21 (1995): 383–386. 8583288 10.1097/00005176-199511000-00003

[12] M. N. Kosek , G. O. Lee , R. L. Guerrant , et al., “Age and Sex Normalization of Intestinal Permeability Measures for the Improved Assessment of Enteropathy in Infancy and Early Childhood,” Journal of Pediatric Gastroenterology and Nutrition 65 (2017): 31–39. 28644347 10.1097/MPG.0000000000001610

[13] L. T. Weaver , M. F. Laker , and R. Nelson , “Intestinal Permeability in the Newborn,” Archives of Disease in Childhood 59 (1984): 236–241. 6424583 10.1136/adc.59.3.236 PMC1628529

[14] E. Roura , S.‐J. Koopmans , J.‐P. Lallès , et al., “Critical Review Evaluating the Pig as a Model for Human Nutritional Physiology,” Nutrition Research Reviews 29 (2016): 60–90. 27176552 10.1017/S0954422416000020

[15] Q. Zhang , G. Widmer , and S. Tzipori , “A Pig Model of the Human Gastrointestinal Tract,” Gut Microbes 4 (2013): 193–200. 23549377 10.4161/gmic.23867 PMC3669164

[16] A. T. Mudd and R. N. Dilger , “Early‐Life Nutrition and Neurodevelopment: Use of the Piglet as a Translational Model,” Advances in Nutrition 8 (2017): 92–104. 28096130 10.3945/an.116.013243 PMC5227977

[17] A. J. Darragh and P. J. Moughan , “The Three‐Week‐Old Piglet as a Model Animal for Studying Protein Digestion in Human Infants,” Journal of Pediatric Gastroenterology and Nutrition 21 (1995): 387–393. 8583289 10.1097/00005176-199511000-00004

[18] H. M. Berschneider , “Development of Normal Cultured Small Intestinal Epithelial Cell Lines Which Transport Na and Cl,” Gastroenterology 96 (1989): A41.

[19] M. M. Geens and T. A. Niewold , “Optimizing Culture Conditions of a Porcine Epithelial Cell Line IPEC‐J2 Through A Histological and Physiological Characterization,” Cytotechnology 63 (2011): 415–423. 21626283 10.1007/s10616-011-9362-9 PMC3140839

[20] C. Nossol , A. Barta‐Böszörményi , S. Kahlert , et al., “Comparing Two Intestinal Porcine Epithelial Cell Lines (IPECs): Morphological Differentiation, Function and Metabolism,” PLoS ONE 10 (2015): 0132323. 10.1371/journal.pone.0132323 PMC4493080 26147118

[21] P. Schierack , M. Nordhoff , M. Pollmann , et al., “Characterization of a Porcine Intestinal Epithelial Cell Line for In Vitro Studies of Microbial Pathogenesis in Swine,” Histochemistry and Cell Biology 125 (2006): 293–305. 16215741 10.1007/s00418-005-0067-z

[22] S. S. Zakrzewski , J. F. Richter , S. M. Krug , et al., “Improved Cell Line IPEC‐J2, Characterized as a Model for Porcine Jejunal Epithelium,” PLoS ONE 8 (2013): 79643. 10.1371/journal.pone.0079643 PMC3829867 24260272

[23] A. J. Brosnahan and D. R. Brown , “Porcine IPEC‐J2 Intestinal Epithelial Cells in Microbiological Investigations,” Veterinary Microbiology 156 (2012): 229–237. 22074860 10.1016/j.vetmic.2011.10.017 PMC3289732

[24] F. Liu , G. Li , K. Wen , et al., “Porcine Small Intestinal Epithelial Cell Line (IPEC‐J2) of Rotavirus Infection As a New Model for the Study of Innate Immune Responses to Rotaviruses and Probiotics,” Viral Immunology 23 (2010): 135–149. 20373994 10.1089/vim.2009.0088 PMC2883522

[25] Z. Chen , L. Zhou , Q. Yuan , H. Chen , H. Lei , and J. Su , “Exosomal miR‐512‐3p Derived From Mesenchymal Stem Cells Inhibits Oxidized Low‐Density Lipoprotein‐Induced Vascular Endothelial Cells Dysfunction via Regulating Keap1,” Journal of Biochemical and Molecular Toxicology 35 (2021): 1–11, 10.1002/jbt.22706. 33760324

[26] P. Liao , M. Liao , L. Li , B. Tan , and Y. Yin , “Effect of Deoxynivalenol on Apoptosis, Barrier Function, and Expression Levels of Genes Involved in Nutrient Transport, Mitochondrial Biogenesis and Function in IPEC‐J2 Cells,” Toxicology Research 6 (2017): 866–877. 30090549 10.1039/c7tx00202e PMC6060741

[27] X. Tang and K. Xiong , “Epidermal Growth Factor Activates EGFR/AMPK Signalling to Up‐Regulate the Expression of SGLT1 and GLUT2 to Promote Intestinal Glucose absorption in Lipopolysaccharide Challenged IPEC‐J2 Cells and Piglets,” Italian Journal of Animal Science 21 (2022): 943–954.

[28] M. Xu , L. Che , L. Niu , et al., “Molecular Mechanism of Valine and its Metabolite in Improving Triglyceride Synthesis of Porcine Intestinal Epithelial Cells,” Scientific Reports 13 (2023): 2933, 10.1038/s41598-023-30036-w. 36806358 PMC9941501

[29] V. Mariani , S. Palermo , S. Fiorentini , A. Lanubile , and E. Giuffra , “Gene Expression Study of Two Widely Used Pig Intestinal Epithelial Cell Lines: IPEC‐J2 and IPI‐2I,” Veterinary Immunology and Immunopathology 131 (2009): 278–284. 19446887 10.1016/j.vetimm.2009.04.006

[30] C. Bernardini , C. Algieri , L. D. Mantia , et al., “Relationship Between Serum Concentration, Functional Parameters and Cell Bioenergetics in IPEC‐J2 Cell Line,” Histochemistry and Cell Biology 156 (2021): 59–67. 33725198 10.1007/s00418-021-01981-2

[31] M. G. Vander Heiden , L. C. Cantley , and C. B. Thompson , “Understanding the Warburg Effect: The Metabolic Requirements of Cell Proliferation,” Science 324 (2009): 1029–1033. 19460998 10.1126/science.1160809 PMC2849637

[32] Z. Hu , Y. Li , H. Du , et al., “Transcriptome Analysis Reveals Modulation of The Stat Family in Pedv‐Infected IPEC‐J2 Cells,” BMC Genomics 21 (2020): 891, 10.1186/s12864-020-07306-2. 33317444 PMC7734901

[33] S. Lu , M. Huffman , Y. Yao , et al., “Regulation of MTP Expression in Developing Swine,” Journal of Lipid Research 43 (2002): 1303–1311. 12177174

[34] K. D. Boger , A. E. Sheridan , A. L. Ziegler , and A. T. Blikslager , “Mechanisms and Modeling of Wound Repair in the Intestinal Epithelium,” Tissue Barriers 11 (2023): 2087454. 35695206 10.1080/21688370.2022.2087454 PMC10161961

[35] M.‐H. Perruchot , G. Boudry , F. Mayeur‐Nickel , et al., “In Vitro Evaluation of Intestinal Barrier Function After Exposure to Digested Pea Ingredients─Food Matrix Effect,” Journal of Agricultural and Food Chemistry 73 (2025): 584–594. 39681414 10.1021/acs.jafc.4c09963 PMC11726683

[36] Y. Chen , H. Rooney , C. Dold , et al., “Membrane Filtration Processing of Infant Milk Formula Alters Protein Digestion in Young Pigs,” Food Research International 166 (2023): 112577. 36914340 10.1016/j.foodres.2023.112577

[37] C. A. Dold , S. L. Bavaro , Y. Chen , et al., “Infant Milk Formula, Produced by Membrane Filtration, Promotes Mucus Production in the Upper Small Intestine of Young Pigs,” Food Research International 187 (2024): 114343. 38763636 10.1016/j.foodres.2024.114343

[38] K. Felix , S. Tobias , H. Jan , S. Nicolas , and M. Michael , “Measurements of Transepithelial Electrical Resistance (TEER) are Affected by Junctional Length in Immature Epithelial Monolayers,” Histochemistry and Cell Biology 156 (2021): 609–616, 10.1007/s00418-021-02026-4. 34459960 PMC8695537

[39] H. Matsumoto , R. H. Erickson , J. R. Gum , M. Yoshioka , E. Gum , and Y. S. Kim , “Biosynthesis of Alkaline Phosphatase During Differentiation of the Human Colon Cancer Cell Line Caco‐2,” Gastroenterology 98 (1990): 1199–1207. 2323513 10.1016/0016-5085(90)90334-w

[40] M. Grauso , A. Lan , M. Andriamihaja , F. Bouillaud , and F. Blachier , “Hyperosmolar Environment and Intestinal Epithelial Cells: Impact on Mitochondrial Oxygen Consumption, Proliferation, and Barrier Function In Vitro,” Scientific Reports 9 (2019): 11360. 31388052 10.1038/s41598-019-47851-9 PMC6684637

[41] V. Cifarelli and N. A. Abumrad in Comprehensive Physiology, ed. R. Terjung (Wiley, 2018), 493–507. 10.1002/cphy.c170026 PMC6247794 29687890

[42] L. V. Gromova , S. O. Fetissov , and A. A. Gruzdkov , “Mechanisms of Glucose Absorption in the Small Intestine in Health and Metabolic Diseases and Their Role in Appetite Regulation,” Nutrients 13 (2021): 2474. 34371983 10.3390/nu13072474 PMC8308647

[43] S. Bröer and S. J. Fairweather , “Amino Acid Transport Across the Mammalian Intestine,” Comprehensive Physiology 9 (2019): 343–373. 10.1002/cphy.c170041 30549024

[44] B. Spanier and F. Rohm in Comprehensive Physiology, ed. R. Terjung (Wiley, 2018), 843–869. 10.1002/cphy.c170038 29687907

[45] C. Andrews , M. H. McLean , and S. K. Durum , “Cytokine Tuning of Intestinal Epithelial Function,” Frontiers in immunology 9 (2018): 1270. 29922293 10.3389/fimmu.2018.01270 PMC5996247

[46] G. Rindi , A. B. Leiter , A. S. Kopin , C. Bordi , and E. Solcia , “The “Normal” Endocrine Cell of the Gut: Changing Concepts and New Evidences,” Annals of the New York Academy of Sciences 1014 (2004): 1–12. 15153415 10.1196/annals.1294.001

[47] B. O. Fabriek , C. D. Dijkstra , and T. K. Van Den Berg , “The Macrophage Scavenger Receptor CD163,” Immunobiology 210 (2005): 153–160. 16164022 10.1016/j.imbio.2005.05.010

[48] M. L. Hermiston , Z. Xu , and A. Weiss , “CD45: A Critical Regulator of Signaling Thresholds in Immune Cells,” Annual Review of Immunology 21 (2003): 107–137. 10.1146/annurev.immunol.21.120601.140946 12414720

[49] F. Fei , J. Qu , C. Li , X. Wang , Y. Li , and S. Zhang , “Role of metastasis‐induced protein S100A4 in Human Non‐Tumor Pathophysiologies,” Cell & Bioscience 7 (2017): 64, 10.1186/s13578-017-0191-1. 29204268 PMC5702147

[50] J. P. Gleeson , K. C. Fein , N. Chaudhary , R. Doerfler , A. N. Newby , and K. A. Whitehead , “The Enhanced Intestinal Permeability of Infant Mice Enables Oral Protein and Macromolecular Absorption Without Delivery Technology,” International Journal of Pharmaceutics 593 (2021): 120120. 33249250 10.1016/j.ijpharm.2020.120120 PMC7790917

[51] L. González‐Mariscal , A. Betanzos , P. Nava , and B. E. Jaramillo , “Tight Junction Proteins,” Progress in Biophysics and Molecular Biology 81 (2003): 1–44. 12475568 10.1016/s0079-6107(02)00037-8

[52] A. Kondrashina , E. Arranz , A. Cilla , et al., “Coupling In Vitro Food Digestion With In Vitro Epithelial Absorption; Recommendations for Biocompatibility,” Critical Reviews in Food Science and Nutrition 64 (2024): 9618–9636. 37233192 10.1080/10408398.2023.2214628

[53] L. Saaby , H. C. C. Helms , and B. Brodin , “IPEC‐J2 MDR1, a Novel High‐Resistance Cell Line With Functional Expression of Human P‐Glycoprotein (ABCB1) for Drug Screening Studies,” Molecular Pharmaceutics 13 (2016): 640–652. 26651362 10.1021/acs.molpharmaceut.5b00874

[54] H. Aly , N. Rohatgi , C. A. Marshall , et al., “A Novel Strategy to Increase the Proliferative Potential of Adult Human β‐Cells While Maintaining Their Differentiated Phenotype,” PLoS ONE 8 (2013): 66131. 10.1371/journal.pone.0066131 PMC3680388 23776620

[55] D. Sharma , M. Singh , and R. Rani , “Role of LDH in Tumor Glycolysis: Regulation of LDHA by Small Molecules for Cancer Therapeutics,” Seminars in Cancer Biology 87 (2022): 184–195. 36371026 10.1016/j.semcancer.2022.11.007

[56] A. C. F. Støy , P. M. H. Heegaard , P. T. Sangild , M. V. Østergaard , and K. Skovgaard , “Gene Expression Analysis of the IPEC‐J2 Cell Line: A Simple Model for the Inflammation‐Sensitive Preterm Intestine,” ISRN Genomics 2013 (2013): 980651.

[57] J. de Vos , R. P. M. A. Crooijmans , M. F. L. Derks , et al., “Comparison of Human Duodenum and Caco‐2 Gene Expression Profiles for 12,000 Gene Sequences Tags and Correlation with Permeability of 26 Drugs,” iScience 26 (2023): 106252. 12425456 10.1023/a:1020483911355

[58] D. Sun , H. Lennernas , L. S. Welage , et al., “Detailed Molecular and Epigenetic Characterization of the Pig IPEC‐J2 and Chicken SL‐29 Cell Lines,” Pharmaceutical Research 19 (2002): 1400–1416.12425456

[59] M. Revilla , A. Puig‐Oliveras , D. Crespo‐Piazuelo , et al., “Expression Analysis of Candidate Genes for Fatty Acid Composition in Adipose Tissue and Identification of Regulatory Regions,” Scientific Reports 8 (2018): 2045, 10.1038/s41598-018-20473-3. 29391556 PMC5794915

