# Supplementary material for: Culture Optimization of the IPEC–J2 Piglet Jejunum Cell Line for Applications in Infant Nutrition Research
Source: Mol Nutr Food Res. 2026 Jun 25;70(12):e70540. doi: 10.1002/mnfr.70540 (PMC13305343; doi:10.1002/mnfr.70540)
Supplement: Supplementary file 1 — Supporting File: mnfr70540‐sup‐0001‐SuppMat.docx. [file MNFR-70-e70540-s001.docx]

**Supplementary Fig. 1:** **Tracking protein digestion during simulated gastrointestinal digestion of dairy infant formula (IF).**


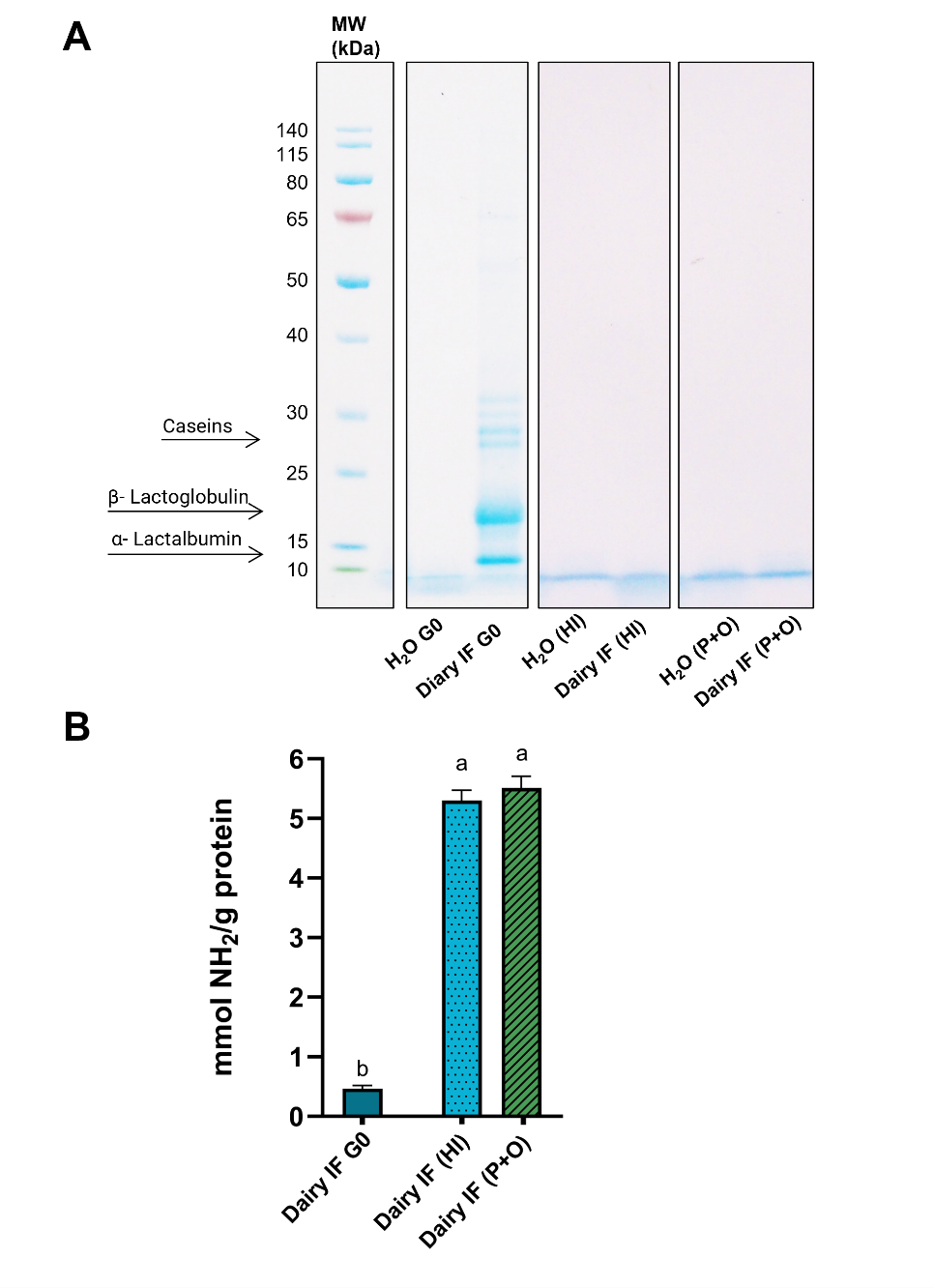
(A) SDS–PAGE profiles of dairy IF before digestion (G0) and after GI digestion, followed by enzyme inactivation by either heat (HI) or Pefabloc + Orlistat (P+O). Each lane contained 10 µg of total protein, separated on a NuPAGE™ 10% Bis–Tris gel under reducing conditions using MOPS running buffer. (B) Degree of protein hydrolysis in dairy IF-G0 (pre-gastric phase) and digested samples (HI and P+O), determined by o-phthaldialdehyde (OPA) assay and expressed as free amine concentration (µmol NH₂/g protein). Protein concentration was determined by Bicinchoninic Acid (BCA) Data represent mean ± SEM from three independent digestions (two technical replicates each). Statistical analysis was performed by one-way ANOVA with Tukey’s post hoc test; bars with different letters differ significantly (P < 0.05).

**Supplementary Fig. 2****: Free amino acid (AA) levels in IPEC-J2 monolayers exposed to digested dairy infant formula (IF) or HBSS.**
IPEC-J2 cells were cultured for 14 days in either PS10 or PS5+EGF+ITS medium and exposed for 2 hours to dairy IF digesta inactivated by heat (HI; 200 µg protein/cm²) or by enzymatic inhibition using Pefabloc and Orlistat (P+O; 150 µg protein/cm²) or to HBSS. (A) Apical free AA concentrations after incubation with dairy IF digesta. (B) Apical-to-basolateral ratios illustrating AA distribution. Color code: blue = higher ratio (greater relative basolateral abundance), white = 0. (C) Basolateral free amino AA concentrations in HBSS-treated monolayers. (D) Apical free AA concentrations in HBSS-treated monolayers. Color code: red = branched-chain AAs (BCAA), orange/red = essential AAs (EAA), and blue/green = non-essential AAs (NEAA). Cya refers to cysteic acid. Data represent mean ± SEM from three independent experiments. Statistical analysis was performed using unpaired Student’s t-tests (A, C, D) or two-way ANOVA with P-values adjusted for multiple testing using the Benjamini–Hochberg false discovery rate method (B). Asterisks indicate significant differences (P < 0.05) between PS5+EGF+ITS and PS10 within each digesta condition, while ‘ns’ denotes not significant.


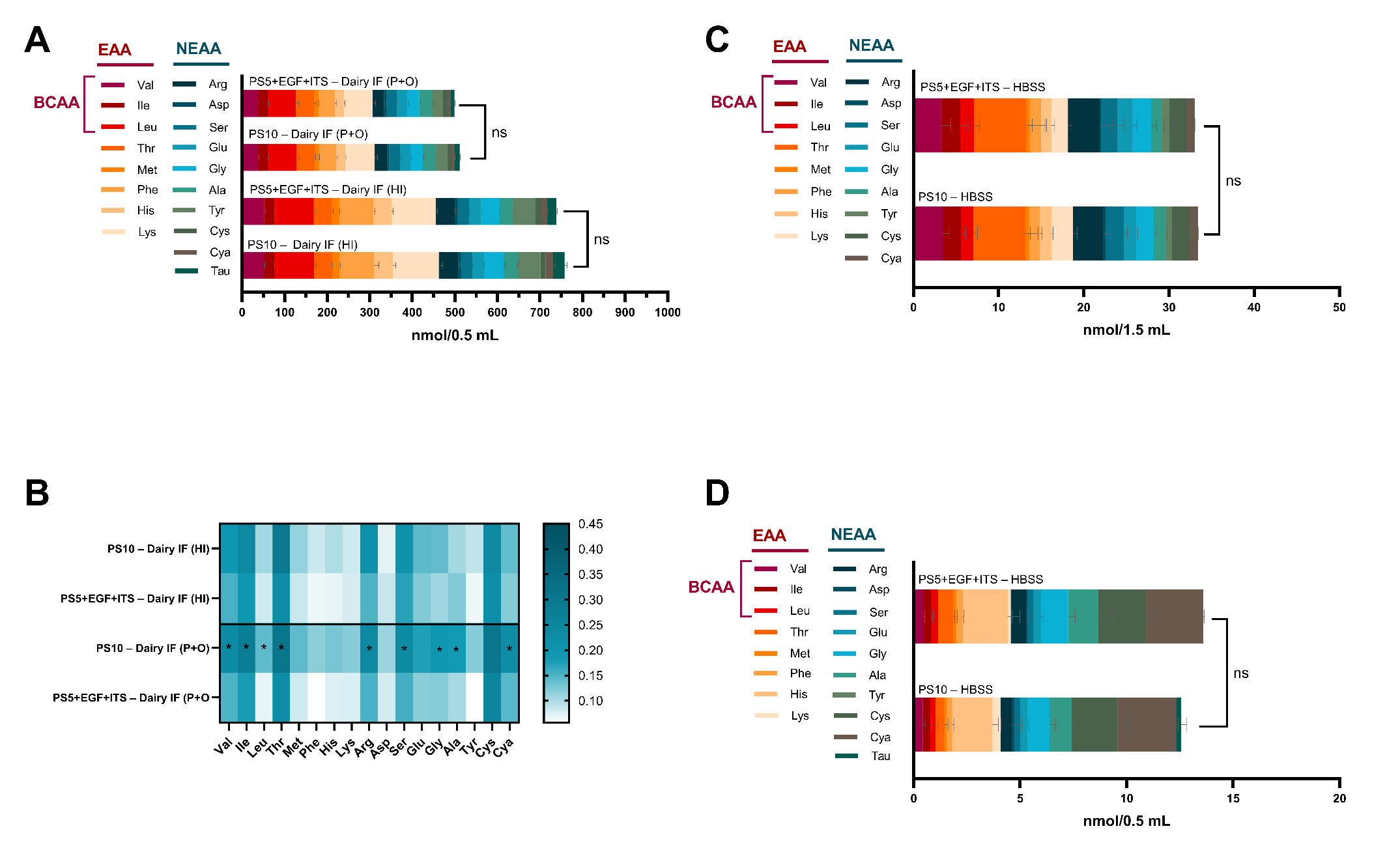


**Supplementary Table 1: (A) Ingredients and (B) nutrient composition of pig diet (on air dry basis as fed).**

| **A** | | **B** | |
| --- | --- | --- | --- |
| **Ingredient** | **Amount (g/kg)** | **Nutrient** | **Amount (g/kg)** |
| Maize (corn) | 260.68 | Dry matter^4^ | 910.5 |
| Lactoflo^1^ | 200 | Moisture^5^ | 89.5 |
| Soybean meal (high protein) | 170.49 | Crude protein^4^ | 202.7 |
| Skimmed milk powder | 125 | Crude fat^4^ | 115.8 |
| Full-fat soybean | 100 | Ash^4^ | 62.7 |
| Soybean oil | 83.5 | Crude fibre^5^ | 18.6 |
| Barley | 24.24 | Total carbohydrates (starch + sugar)^5^ | 431.7 |
| Limestone flour | 7 | Sugars^5^ | 251.9 |
| Lysine HCl (78.8) | 6.09 | Starch^5^ | 179.8 |
| Monocalcium/dicalcium phosphate | 5 | Neutral detergent fibre (NDF) ^4^ | 57.8 |
| L-Threonine (98) | 3.6 | Acid detergent fibre (ADF) ^5^ | 22.7 |
| DL-Methionine | 3.6 | Digestible energy (DE) ^5^ | 16.23 (MJ/kg) |
| Vitamin–mineral premix (weaner)^2^ | 3 | Net energy (NE) ^4^ | 12.28 (MJ/kg) |
| Salt feed grade | 3 | Total lysine^4^ | 16.19 |
| L-Valine | 2.3 | Standardised ileal digestible lysine^5^ | 15.3 |
| L-Tryptophan | 1.4 |  | |
| Sepiolite | 0.1 |  |  |
| Phytase enzyme^3^ | 0.1 |  |  |

^1^ Lactoflo is a whey-derived lactose-rich ingredient (Volac, UK) containing approximately 82% lactose, 3.5% protein, 8% ash, 5% moisture, and 0.1% fat.

^2^ Vitamin and mineral premix provided per kilogram of complete diet: Cu from copper sulphate, 15mg; Fe from ferrous sulphate monohydrate, 90 mg; Zn from zinc oxide, 120 mg; Mn from manganese oxide, 47 mg; Ca from calcium iodate, 0.6 mg; Se from sodium selenite, 0.3 mg; vitamin A as retinyl acetate, 1.5 mg; vitamin D_3_ as cholecalciferol, 12.5 µg; vitamin E as DL-alpha-tocopheryl acetate, 90 mg; vitamin K, 4 mg; vitamin B_12_, 15 µg; riboflavin, 2 mg; nicotinic acid, 12 mg; pantothenic aid, 10 mg; choline chloride, 250 mg; vitamin B_1_, 2 mg; and vitamin B_6_, 3 mg.

^3^ The diets contained 500 phytase units (FTU) per kilogram feed from RONOZYME HiPhos (DSM, Belfast, UK).

^4^ Analysed values.

^5^ Calculated values.

**Supplementary Table 2: Gene name, protein name, protein function, GenBank accession number, primers sequences and annealing temperature (°C) for RT-PCR analysis.**

| **Gene Symbol** | **Protein Name** | **Function** | **Accession Number** | **Forward Primer** | **Reverse Primer** | **Annealing Temperature (°C)** | **Source** |
| --- | --- | --- | --- | --- | --- | --- | --- |
| *RPLP0* | 60S acidic ribosomal protein P0 | Housekeeping gene | NM_001098598.1 | GACAAAGTGGGAGCCAGTGA | CAGGGTTGTAGATGCTGCCA | 56 | [57] |
| *MUC1* | Mucin-1 (MUC1) | Membrane-bound mucin | XM_021089729.1 | AGTCCATGTTGGCACCTCC | GGAGTACCTTTGCTGACTGG | 55 | [57] |
| *MUC2* | Mucin-2 (MUC2) | Secreted gel-forming mucin | XM_013989745.1 | TCTGGATCCGCAGCTCTCTGG | CACTGGGCTGGGAGACAGGT | 60 | [57] |
| *CLDN2* | Claudin-2 (CLDN2) | Tight junction protein—paracellular cation-selective channels | NM_001244539.1 | ACCGTGTGGGAACAACCAGA | ACACATGAAAATGGCTTCCCTCC | 62 | [57] |
| *CLDN1* | Claudin-1 (CLDN1) | Tight junction protein—barrier integrity | NM_001161638.1 | GCATCATTTCCTCCCTGTT | TCTTGGCTTTGGGTGGTT | 60 | [57] |
| *CLDN4* | Claudin-4 (CLDN4) | Tight junction protein—barrier integrity | NM_001161637.1 | CAACTGCGTGGATGATGAGA | CCAGGGGATTGTAGAAGTCG | 60 | [57] |
| *OCLN* | Occludin (OCLN) | Tight junction protein—barrier integrity | NM_001163647.2 | TCCTGGGTGTGATGGTGTTC | CGTAGAGTCCAGTCACCGCA | 63 | [57] |
| *TJP1* | Zonula occludens 1 (ZO1) | Tight junction scaffold protein | XM_003353439.2 | ATGGCGGAAAGTGAACCTCG | TCACACCCTGCTTAGAATCCG | 55 | [57] |
| *IL6* | Interleukin-6 (IL-6) | Cytokine | NM_214399.1 | TGGCTACTGCCTTCCCTACC | CAGAGATTTTGCCGAGGATG | 60 | [57] |
| *IL8* | Interleukin-8 (IL8) | Chemokine | NM_213867.1 | GAAGAGAACTGAGAAGCAACAACA | TTGTGTTGGCATCTTTACTGAGA | 60 | [57] |
| *TNF* | Tumor necrosis factor (TNF) | Cytokine | NM_214022.1 | CACGTTGTAGCCAATGTCAAAG | GAGGTACAGCCCATCTGTCG | 60 | [57] |
| *CD163* | Cluster of differentiation 163 (CD163) | Macrophage scavenger receptor | NM_213976.1 | CACATGTGCCAACAAAATAAGAC | CACCACCTGAGCATCTTCAA | 60 | [54] |
| *S100A4* | S100 calcium-binding protein A4 (S100A4/FSP1) | Fibroblast marker | NM_001252605.1 | GGCCCTCGATGTGATGGTGT | CATCCGTCCTTTTCCCCAGGA | 60 | This study |
| *CHGA* | Chromogranin A (CHGA) | Enteroendocrine cell marker | NM_001164005.2 | GCAAGTCATTGCCCTCCCTG | GAGGATCCGTTCATCTCCTCGG | 60 | This study |
| *LGR5* | Leucine-rich repeat-containing G protein-coupled receptor 5 (LGR5) | Intestinal stem cell marker | NM_001315762.1 | GCCTTTGTAGGCAACCCTTCT | AGGCACCATTCAAAGTCAGTGT | 60 | This study |
| *PTPRC* | Protein tyrosine phosphatase receptor type C (CD45) | Leukocyte marker | XM_003130596 | AAAGCTCGGCCAGGAATGGT | TGGGGCACTTGGTGAACTGA | 60 | This study |
| *SLC5A1* | Sodium/glucose cotransporter 1 (SGLT1) | Na⁺/glucose co-transporter (apical) | NM_001098598.1 | CTGCAAGAGAGTCAATGAGGAG | CCGGTTCCATAGGCAAACT | 60 | [50] |
| *SLC2A2* | Glucose transporter 2 (GLUT2) | Facilitative glucose transporter (basolateral) | NM_001097417 | ACGCAACCATTGGAGTTGGC | AGCACAAGTCCCACCGACAT | 60 | This study |
| *SLC27A4* | Fatty acid transport protein 4 (FATP4) | Long-chain fatty acid transporter and acyl-CoA synthetase (apical) | XM_021069609.1 | GCTGCATAAAACAGGGACTTTCA | AACAGCGGGTCTTTCACGACT | 60 | [58] |
| *CD36* | Cluster of differentiation 36 (CD36/FAT) | Fatty acid translocase (apical) | NM_001044622.1 | GGTCCTTACACGTACAGAGTTCGTT | CCATTGGGCTGTAGGAAAGAGA | 60 | [58] |
| *SLC15A1* | Peptide transporter 1 (PEPT1) | Peptide transporter (apical) | NM_214347.1 | GGCTTCCATGGCTTTCGTGG | TCAAGGGTCACCGTCGTTCC | 60 | This study |
| *SLC6A20* | IMINO | Apical uptake of proline and imino acids | XM_021068640.1 | TCGTGTCCCTCATCAACAG | AGGAAGCCATCTTCAAGGTC | 60 | This study |
| *SLC6A19* | Neutral amino acid transporter B0AT1 (B0AT1) | Apical uptake of neutral amino acids (e.g. Leu, Val) | XM_003359855.4 | CTTTCATCTTCACCCTGAACTC | GATGTCGCTGTTGAACCTG | 60 | This study |
| *SLC3A1* | Related to b0,+ amino acid transporter (rBAT) | Apical transporter of cystine and basic AA transport) | NM_001123042.1 | CAATGCAGTGGGACAACAG | GGCGTGAAGCAAACTTAATTC | 60 | This study |
| *SLC6A14* | Amino acid transporter B0,+ (ATB⁰,+) | Apical uptake of neutral and cationic amino acids | NM_001348402.1 | CTGTGGCTTGGGGTGGTTTA | AACCAAGCAGCAACCCAAAG | 60 | This study |
| *SLC1A5* | Alanine–serine–cysteine transporter 2 (ASCT2) | (ASCT2)  Apical uptake of neutral amino acids (e.g. Ala, Ser, Gln) | NM_001444293.1 | CGATTCGTTCCTGGA TCTTG | TAGGACGTCGCGTATGAG | 60 | This study |
| *SLC7A8* | L-type amino acid transporter 2 (LAT2) | Basolateral transport of neutral amino acids | XM_021099239.1 | ACTACCTCTTCTATGGCATCAC | GCAAGTAGATGATGGGGAACAG | 60 | This study |
| *SLC7A7* | y⁺L amino acid transporter 1 (y⁺LAT1) | Basolateral exchange of cationic and neutral amino acids (basolateral) | [NM_001110421](http://www.ncbi.nlm.nih.gov/nuccore/NM_001110421) | CTCTGCTGTTCAATGGTCTC | ATAGAGCTGACCCACGATAG | 60 | This study |
| *SLC43A2* | L-type amino acid transporter 4 (LAT4) | Basolateral efflux of neutral amino acids | XM_021067563.1 | CAGATCCAGAAGATCACCAAC | TGAAGGAGAGAATCTGTAGGG | 60 | This study |

**Supplementary Table 3:** **mRNA expression in porcine jejunal mucosal scrapings relative to IPEC-J2 monolayers cultured in FBS10**.

Messenger RNA was quantified by RT–qPCR, normalised to *RPLP0*, and expressed as fold change using the 2^−ΔΔCt^ method (FBS10 = 1). Values are reported as geometric mean (2^−ΔΔCt^) with 95% CI. p-values correspond to the jejunum vs FBS10 comparison from a Student’s t-test performed on ΔΔCt (log₂) values. Significance: *P < 0.05, **P < 0.01, ***P < 0.001, ****P < 0.0001 (n = 4 biologically independent samples; 2 female pigs, 2 male pigs, age 56 days).

| **Gene** | **Common name** | **Fold change (FBS10 =1)** | **SEM** | **p-value (vs. FBS10)** | **Significance** |
| --- | --- | --- | --- | --- | --- |
| *S100A4* | S100 calcium-binding protein A4 (S100A4/FSP1) | 0.03 | 0.01 | 0.0002 | *** |
| *SLC15A1* | Peptide transporter 1 (PEPT1) | 220011.48 | 37753.33 | <0.0001 | **** |
| *SLC5A1* | Sodium/glucose cotransporter 1 (SGLT1) | 14628.12 | 2766.30 | <0.0001 | **** |
| *SLC2A2* | Glucose transporter 2 (GLUT2) | 3.06 | 0.57 | 0.0115 | * |
| *SLC27A4* | Fatty acid transport protein 4 (FATP4) | 307.64 | 35.33 | <0.0001 | **** |
| *CD36* | Cluster of differentiation 36 (CD36/FAT) | 14.79 | 6.10 | 0.0019 | ** |
| *LAT2* | L-type amino acid transporter 2 (LAT2) | 153074.28 | 45294.51 | <0.0001 | **** |
| *LAT4* | L-type amino acid transporter 4 (LAT4) | 1150.03 | 200.94 | <0.0001 | **** |
| *rBAT* | Related to b0,+ amino acid transporter (rBAT) | 45.49 | 10.32 | <0.0001 | **** |
| *ASCT2* | Alanine–serine–cysteine transporter 2 (ASCT2) | 1.52 | 0.43 | 0.4329 | ns |
| *SLC7A7* | y⁺L amino acid transporter 1 (y⁺LAT1) | 13.50 | 5.25 | 0.0024 | ** |
| *OCLN* | Occludin (OCLN) | 2.14 | 0.32 | 0.0176 | * |
| *TJP1* | Zonula occludens 1 (ZO1) | 0.41 | 0.04 | 0.0010 | ** |
| *CLDN2* | Claudin-2 (CLDN2) | 389089.69 | 112159.24 | <0.0001 | **** |
| *CLDN1* | Claudin-1 (CLDN1) | 0.01 | 0.01 | 0.0012 | ** |
| *CLDN4* | Claudin-4 (CLDN4) | 4.68 | 0.89 | 0.0057 | ** |
| *IL6* | Interleukin-6 (IL-6) | 0.16 | 0.05 | 0.0048 | ** |
| *IL8* | Interleukin-8 (IL8) | 6.44 | 1.80 | 0.0022 | ** |
| *TNF* | Tumor necrosis factor (TNF) | 77.55 | 8.38 | <0.0001 | **** |
| *MUC1* | Mucin-1 (MUC1) | 0.02 | 0.01 | <0.0001 | **** |
| *MUC2* | Mucin-2 (MUC2) | 1212.78 | 679.12 | 0.0001 | *** |

**Supplementary Table 4:** **Free amino acid concentrations in dairy infant formula (IF) digesta**. Free amino acid (AA) concentrations (mean and SEM, nmol/mL) in dairy IF digesta detoxified by either heat (HI) or by Pefabloc+Orlistat (P+O). Samples were analyzed in 3 independent digestions (with two technical replicates each).

| **Amino acid** | **Digesta - Dairy IF (HI)** | | **Digesta - Dairy IF (P+O)** | |
| --- | --- | --- | --- | --- |
|  | **Mean (nmol/mL)** | **SEM** | **Mean (nmol/1.5 mL)** | **SEM** |
| Val | 999.27 | 55.74 | 1191.16 | 96.63 |
| Ile | 463.19 | 48.23 | 611.22 | 78.03 |
| Leu | 1955.019 | 146.03 | 2407.35 | 268.79 |
| Thr | 899.09 | 150.13 | 1222.05 | 264.29 |
| Met | 374.20 | 51.10 | 466.43 | 75.565 |
| Phe | 2001.63 | 204.49 | 1988.09 | 141.82 |
| His | 1051.41 | 131.05 | 1079.07 | 84.7 |
| Lys | 2506.28 | 107.25 | 2659.74 | 62.07 |
| Arg | 982.51 | 84.04 | 1063.41 | 50.95 |
| Asp | 196.51 | 8.23 | 237.89 | 24.58 |
| Ser | 541.85 | 97.43 | 738.73 | 174.07 |
| Glu | 672.19 | 50.44 | 889.58 | 132.39 |
| Gly | 1087.77 | 58.08 | 1101.29 | 41.20 |
| Ala | 631.57 | 121.63 | 851.25 | 191.98 |
| Tyr | 1245.85 | 80.81 | 1293.38 | 12.08 |
| Cys | 290.39 | 4.97 | 321.15 | 8.65 |
| Cya | 310.57 | 47.13 | 276.36 | 30.36 |
| Tau | 557.94 | 69.18 | 518.70 | 16.98 |

**Supplementary Table** **5: Free amino acid concentrations in the basolateral chamber of IPEC-J2 monolayers exposed to dairy IF digesta**. Basolateral free amino acid (AA) concentrations (mean ± SEM, nmol/mL) in IPEC-J2 monolayers cultured for 14 days in either PS10 or PS5 + EGF + ITS medium and exposed for 2 h to dairy IF digesta inactivated by heat (HI; 200 µg protein/cm²) or Pefabloc+Orlistat (P+O; 150 µg protein/cm²). Data represent three independent experiments (two technical replicates each).

| **Amino acid** | **PS10 – Diary IF (HI)** | | **PS5+EGF+ITS – Diary IF (HI)** | | **PS10 – Diary IF (P+O)** | | **PS5+EGF+ITS – Diary IF (P+O)** | |
| --- | --- | --- | --- | --- | --- | --- | --- | --- |
|  | **Mean (nmol/mL)** | **SEM** | **Mean (nmol/mL)** | **SEM** | **Mean (nmol/mL)** | **SEM** | **Mean (nmol/mL)** | **SEM** |
| Val | 9.63 | 0.98 | 7.90 | 2.00 | 10.02 | 0.39 | 6.59 | 1.04 |
| Ile | 5.79 | 0.51 | 4.55 | 1.54 | 6.28 | 0.21 | 4.02 | 0.96 |
| Leu | 10.68 | 2.17 | 8.22 | 3.41 | 12.49 | 1.43 | 6.21 | 1.86 |
| Thr | 13.62 | 1.70 | 10.43 | 2.61 | 13.91 | 0.91 | 9.87 | 1.46 |
| Met | 2.19 | 0.26 | 1.67 | 0.64 | 2.42 | 0.20 | 1.40 | 0.32 |
| Phe | 8.64 | 2.38 | 6.83 | 1.77 | 8.63 | 1.54 | 4.25 | 0.96 |
| His | 5.36 | 1.27 | 3.70 | 1.04 | 5.07 | 0.60 | 2.99 | 0.41 |
| Lys | 10.51 | 3.07 | 10.14 | 3.19 | 11.71 | 1.43 | 7.47 | 1.38 |
| Arg | 10.80 | 1.01 | 7.57 | 2.14 | 9.37 | 0.35 | 5.97 | 1.19 |
| Asp | 0.73 | 0.10 | 0.87 | 0.19 | 0.97 | 0.10 | 0.70 | 0.07 |
| Ser | 5.68 | 0.46 | 4.77 | 1.22 | 6.30 | 0.14 | 4.22 | 0.76 |
| Glu | 4.93 | 0.48 | 4.21 | 1.38 | 5.69 | 0.31 | 3.86 | 0.37 |
| Gly | 7.78 | 1.06 | 5.97 | 1.16 | 7.71 | 0.65 | 4.90 | 0.63 |
| Ala | 3.42 | 0.42 | 3.99 | 1.39 | 5.67 | 1.08 | 3.24 | 0.89 |
| Tyr | 5.73 | 1.13 | 4.17 | 1.61 | 5.72 | 1.00 | 2.71 | 0.83 |
| Cys | 3.56 | 0.58 | 3.26 | 0.30 | 3.65 | 0.44 | 2.95 | 0.26 |
| Cya | 2.21 | 0.68 | 1.91 | 0.10 | 2.24 | 0.28 | 1.47 | 0.21 |
